# Revised age for Schöningen hunting spears indicates intensification of Neanderthal cooperative behavior around 200,000 years ago

**DOI:** 10.1126/sciadv.adv0752

**Published:** 2025-05-09

**Authors:** Jarod M. Hutson, Felix Bittmann, Peter Fischer, Alejandro García-Moreno, Sabine Gaudzinski-Windheuser, Ellie Nelson, José E. Ortiz, Kirsty E. H. Penkman, Zoran M. Perić, Daniel Richter, Trinidad Torres, Elaine Turner, Aritza Villaluenga, Dustin White, Olaf Jöris

**Affiliations:** ^1^ MONREPOS Archaeological Research Centre and Museum for Human Behavioural Evolution, Leibniz Zentrum für Archäologie, 56567 Neuwied, Germany.; ^2^ Department of Paleobiology, National Museum of Natural History, Smithsonian Institution, Washington, D.C. 20013, USA.; ^3^ Lower Saxony Institute for Historical Coastal Research, 26382 Wilhelmshaven, Germany.; ^4^ Institute of Geography, University of Bremen, 28359 Bremen, Germany.; ^5^ Institute for Geography, Johannes Gutenberg University Mainz, 55099 Mainz, Germany.; ^6^ MUPAC Museum of Prehistory and Archaeology of Cantabria, 39009 Santander, Spain.; ^7^ Institute of Ancient Studies, Department of Prehistoric and Protohistoric Archaeology, Johannes Gutenberg University Mainz, 55099 Mainz, Germany.; ^8^ NEaar Laboratory, Department of Chemistry, University of York, York YO10 5DD, UK.; ^9^ Laboratory of Biomolecular Stratigraphy, E.T.S.I. Minas y Energía, Universidad Politécnica de Madrid, 28003 Madrid, Spain.; ^10^ Department of Geology, Lund University, Sölvegatan 12, SE-223 62 Lund, Sweden.; ^11^ Zentrum für Baltische und Skandinavische Archäologie, Leibniz Zentrum für Archäologie, 24837 Schleswig, Germany.; ^12^ Department of Human Origins, Max Planck Institute for Evolutionary Anthropology, 04103 Leipzig, Germany.; ^13^ Consolidated Research Group on Prehistory: Human Evolution, Climate Change and Cultural Adaptation in Preindustrial Societies (GIZAPRE IT-1435-22), University of the Basque Country (UPV/EHU), 01006 Vitoria-Gasteiz, Spain.; ^14^ Department of Prehistory, Ancient History, and Archaeology, Complutense University of Madrid, 28040 Madrid, Spain.

## Abstract

The Schöningen 13II-4 archaeological site in Germany holds title to the most complete Paleolithic wooden hunting spears ever discovered, yet its age has never been properly settled. Initial estimates placed the site at around 400,000 years; this age was later revised to roughly 300,000 years. Here, we report age estimates for the “Spear Horizon” based on amino acid geochronology of fossils obtained directly from the find-bearing deposits. Together with a reassessment of regional Middle Pleistocene chronostratigraphy, these data place the Schöningen spears at ~200,000 years. This revised age positions the Spear Horizon alongside other sites that collectively record a shift toward communal hunting strategies. The Schöningen archaeological record exemplifies this behavioral transformation that arose within the increasingly complex social environments of Middle Paleolithic Neanderthals.

## Teaser

The world’s oldest complete wooden hunting weapons from Schöningen, Germany, are 100,000 years younger than previously claimed.

## INTRODUCTION

**Fig. 1.**
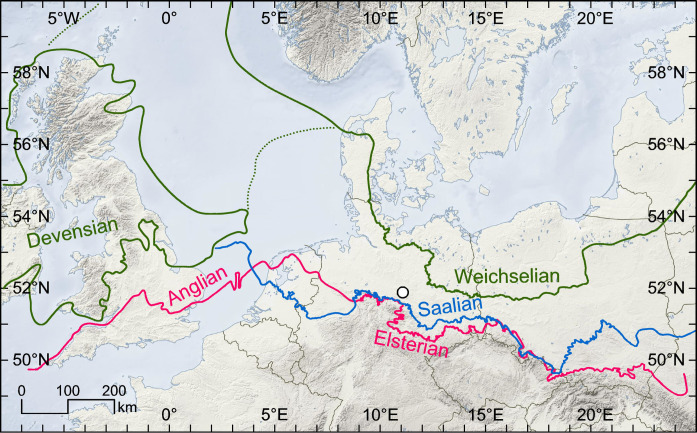
Location of Schöningen. Map showing the location of Schöningen (white dot) relative to the maximum extent of major Middle (Anglian, Elsterian, and Saalian advances) and Upper (Devensian and Weichselian) Pleistocene inland glacial advances across northern Europe (*100*, *101*). Map based on SRTM30_PLUS data.

The Schöningen 13II-4 “Spear Horizon” site (Landkreis Helmstedt, Lower Saxony, Germany) (Fig. 1) lays claim to the world’s oldest complete wooden hunting weapons (*1*), including nine spears, one lance, and six double-pointed sticks (*2*). This arsenal is joined by a small assemblage of about 1500 flint artifacts (*3*) scattered around the butchered remains of more than 50 horses (table S1) (*4*), altogether representing the spoils of repeated ambush hunting episodes along the shoreline of a former lake (*4*–*9*). Nowhere else in the Middle Pleistocene record are such hunting encounters so well preserved in undisturbed contexts. Accordingly, the site has come to serve as a marker horizon in the development of premodern human hunting abilities (*10*). Given this, it is crucial to ascribe an accurate age to the site to place the technologies and behaviors revealed from the Spear Horizon in the broader timeline of human behavioral evolution.

Quaternary exposures at the Schöningen open-cast lignite mine represent some of the most complete terrestrial archives covering the latter half of the Middle Pleistocene (see Supplementary Text and figs. S1 to S3) and contain numerous Paleolithic archaeological localities, most notably the 13II-4 Spear Horizon (*11*). The Spear Horizon itself has never been directly dated successfully. Instead, age estimates for the spears have relied on a generalized correlation of the stratigraphic sequence to the global paleoclimatological record in combination with cross-dating of other fossil levels within the Schöningen mine complex (see Supplementary Text).

The stratigraphic sequence at Schöningen spans from the Elsterian glaciation to the Holocene (Table 1; see also Supplementary Text, figs. S2 and S3, and table S2) (*12*–*14*). The Elsterian represents the southernmost advance of the Fennoscandian ice sheet into northern central Europe and dates to marine oxygen isotope stage (MIS) 12 (*15*–*18*). At Schöningen, Elsterian glacial tills and clays are followed by a series of intersecting sediment-filled channels (Cycles I to VI, from bottom to top and old to young). Channels containing Cycle I to III deposits are argued to have formed during periods of major climatic and environmental change, most likely around the termination of the major glacial cycles, and subsequently filled by deltaic-lacustrine deposits during the ensuing interglacial periods. The basic assumption that roughly 100,000 years (kyr) long interglacial-glacial cycles start and end with major glacial terminations, during which the channels formed, provides the backbone of the relative chronology at Schöningen, but such a strict correlation has not been established directly. This late Middle Pleistocene sequence is sealed by ground moraines of the Drenthe ice advance of the Saalian glaciation (*12*–*14*) dated to MIS 6 (Table 1) (*19*–*21*). Drenthian tills are succeeded by MIS 5e Eemian interglacial deposits in the Schöningen northern mining field (*13*) and by loess-paleosol sequences of Cycles IV to V and Holocene Cycle VI deposits in the southern mining field (*14*).

**Table 1.** Late Middle Pleistocene to Holocene sequence at Schöningen. The Schöningen stratigrapic sequence compared with MISs and regional subdivisions for this period based on the MIS age of interglacial and glacial deposits (see Supplementary Text). The late Middle Pleistocene Schöningen Cycles I to III are bracketed between Elsterian and Drenthian tills of MIS 12 and 6 age. Therefore, Schöningen Cycles I to III are to be placed between MIS 12 and 6, but their correlation with MIS stages remains poorly established and is based on the assumption that the Alversdorf Interglacial of Cycle I correlates to the Holsteinian Interglacial of MIS 11c. Bolded and underlined text shows the position of the Schöningen 13II-4 “Spear Horizon.” LGM, last glacial maximum; IG, interglacial.

Units of the Quaternary	British Isles	Western Central Europe	Schöningen		Eastern Central Europe	MIS
Holocene	Holocene	Holocene	Cycle VI	Holocene	Holocene	1
Upper Pleistocene	LGM advances	LGM advances		Weichselian	LGM advances	2
						3
	Devensian	Weichselian	Cycle VCycle IV		Weichselian	4
						5d-5a
	Ipswichian	Eemian		Eemian	Eemian	5e
Late Middle Pleistocene	*Poorly resolved*	Warthe advance			Warthe advance	6
		Drenthe advance	Drenthian		Oder advance	
		Saalian complex	Cycle III 13 II-4^*^ Cycle II Cycle I	Schöningen IG 13 II-4^*^ Reinsdorf IG Alversdorf IG	*Poorly resolved*	7
						8
						9
						10
	Hoxnian	Holsteinian			Masovian	11c
	Anglian	Elsterian	Elsterian^†^		San-2	12

*The Schöningen 13II-4 Spear Horizon is contained in a late phase of Cycle II deposits [transition from biostratigraphic units BU-C to BU-D (*27*)], several meters above the Reinsdorf Interglacial peak sediments.

†At Schöningen, several forested interstadials of presumably late Elsterian age have been described from the northern mining field, preceding the Alversdorf Interglacial (*12*), which are referred to Cycle 0 in the text.

The Elsterian (MIS 12) and Drenthian (MIS 6) ice advances represent the most severe cold periods of the European Middle Pleistocene (Fig. 1), which brought about substantial landscape remodeling (*16*–*22*). Throughout the northern European Lowlands, smaller depressions left after the retreat of the ice sheets were filled during the succeeding Holsteinian (MIS 11 peak) and Eemian (~MIS 5e) interglacial periods (Table 1; see also tables S2 and S3). With quite uniform vegetational successions, the Holsteinian Interglacial is closely linked to the Hoxnian Interglacial in the British Isles and the Masovian Interglacial known from Poland (Table 1; see also Supplementary Text) (*23*). Similarly, the Eemian of northern Central Europe correlates with the Ipswichian on the British Isles (*24*).

Pollen preserved at different exposures in the Schöningen lignite mining complex between the Elsterian and Drenthian tills shows that most of the deltaic-lacustrine deposits of Cycles I to III predominantly formed under interglacial or interstadial climatic conditions, with the landscape shifting between forested and more open, steppic vegetation. On the basis of their palynology, the peak interglacial phases of Cycles I to III were locally named Alversdorf, Reinsdorf, and Schöningen (*13*, *25*) and are believed to correlate with the warmest periods of MIS 11, 9, and 7, respectively (*13*, *26*–*28*) (Table 1; see also table S2). This correlation forms the basis for the Schöningen standard chronostratigraphic model applied in most recent publications (*27*–*29*). Each of these interglacial phases were succeeded by deposits of forested interstadial character, lacking thermophilous plant species. Similar interstadials, locally named Offleben I, Offleben II, and Esbeck, also preceded the Alversdorf Interglacial and have been assigned a late Elsterian age (*12*). Here, these pre-Alversdorf deposits are referred to as “Cycle 0” (see figs. S2 and S3).

**Fig. 2.**
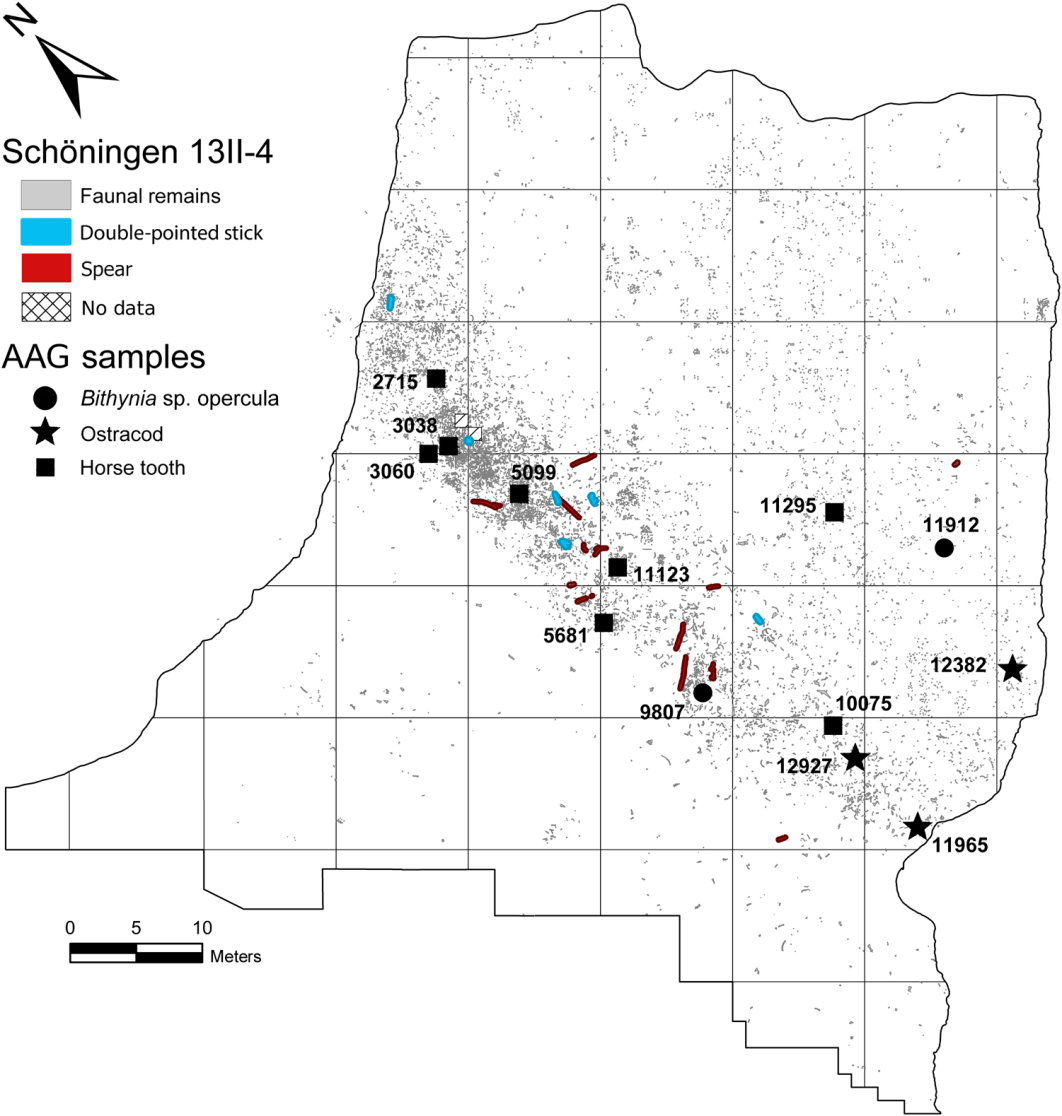
Schöningen 13II-4 Spear Horizon excavation plan. Map showing the locations of opercula, ostracod, and horse tooth samples selected for AAG analysis (sample numbers refer to Table 2 and tables S4 and S5). Four *Bithynia* opercula were analyzed from two loci; 13 ostracod samples were analyzed from three loci; nine horse dentine samples were analyzed from eight individual molars. Stratigraphically, the Spear Horizon within site Schöningen 13II-4 measures some 20 to 40 cm in thickness, encompassing layers 4b (laminated organic silt) and 4b/4c (transition to calcareous silt) (*102*) at the transition from Reinsdorf BU-C to BU-D (*27*). Some archaeological remains from layers 4a (organic silt) and the top of 4c (calcareous silt) are also included in the Spear Horizon. The site has been almost completely excavated, covering some 3900 m^2^ and yielding nearly 15,000 archaeological finds (*11*). Spatially, the main find concentration occupies a 60 m–by–10 m band straddling the North-South–oriented shoreline of the former lake (*68*, *102*–*105*). Note that excavation squares shown are 10 m by 10 m.

Archaeological finds from the Schöningen 13II-4 Spear Horizon originate from layers 4a to 4c within the later part of the Cycle II (Reinsdorf) sequence, at the transition from biostratigraphic unit C to D (BU-C to BU-D) (*27*), formerly assigned to the Reinsdorf-B Interstadial (Fig. 2 and Table 1; see also figs. S2 and S3 and tables S1 and S2). At this time, the local landscape consisted of woodland or open woodland of alder, birch, and willow, with meadows and raised bogs flanking the former lakeshore (*27*). Hominin occupation of the Spear Horizon coincided with the transition from an open woodland steppic phase (layer 4c; BU-C) to the second forested interstadial phase that succeeded the Reinsdorf Interglacial (layers 4a/b; BU-D), presumably close in time to the onset of the next glacial period (*28*, *29*).

Age estimates for the Spear Horizon have been amended over the years from ~400,000 to ~300,000 years ago (ka) (*1*, *30*–*32*), MIS 11 or MIS 9, depending on age estimates for the preceding Cycle I deposits and alternative correlations with isotope climate records (*30*). Thermoluminescence (TL) dating of heated flint from the Schöningen 13I-1 (Cycle I) archaeological site resulted in an age of 321 ± 14 ka, placing the site into MIS 9 (*31*) and the Alversdorf peak interglacial deposits into MIS 9e. The Schöningen 13II-4 Spear Horizon finds come from close to the top of the next younger sedimentological cycle and should therefore date to MIS 7. Uranium-series dates of 290 ± 5 ka for peat horizons within the Cycle II sequence were withdrawn because of their recognized open-system behavior (*33*, *34*). Optically stimulated luminescence (OSL) data assign level 13II-2c1 (Cycle II) a maximum age of ~300 ka (*32*). Level 13II-2c1 formed during cold climatic conditions (BU-A) immediately after the Reinsdorf Interglacial, indicating deposition of the sediment in MIS 9d (*27*, *32*). On the basis of these estimates, both Cycle I and Cycle II sequences would date to MIS 9. The TL and OSL dates from units 13I-1 and 13II-2c1, however, originate from samples located stratigraphically below unit 13II-4 and can therefore only serve as maximum age estimates for the Spear Horizon. Here, we provide age estimates for the Schöningen spears and associated archaeology based on amino acid geochronology (AAG) of fossils recovered directly from the Spear Horizon deposits.

## RESULTS

Systematic sampling for AAG incorporated multiple fossil types recovered from among the densest concentrations of archaeological finds in the Schöningen 13II-4 Spear Horizon (Fig. 2; see also fig. S4). AAG data were obtained using a reverse-phase high-performance liquid chromatograph (RP-HPLC) from the intracrystalline fraction of four *Bithynia tentaculata* opercula (Table 2). As intracrystalline protein decomposition (IcPD) in opercula represents an effective closed system of fossil protein (*35*), these data were the primary means to derive an age estimation. Additional independent numerical AAG age estimates from ostracods and horse teeth (Fig. 2; see also tables S4 and S5) are considered exploratory given the potential open-system nature of the samples and the scarcity of the required comparator material.

**Table 2.** Provenance and amino acid data for Schöningen opercula samples. Error terms represent 1 SD about the mean for the duplicate analyses for an individual sample. Each sample was bleached (b), with the free amino acid (FAA) fraction signified by “F” and the total hydrolysable fraction by “H*” (see Materials and Methods). NEaar, North East Amino Acid Racemization.

Schöningen ID	Provenance	Layer	NEaar no.	Ala D/L	Asx D/L	Glx D/L	Ser D/L	Val D/L
9807	709/2	4b and 4b/4c	10606bF	0.300 ± 0.000	0.708 ± 0.004	0.169 ± 0.006	0.986 ± 0.000	0.170 ± 0.002
			10606bH*	0.262 ± 0.002	0.641 ± 0.000	0.166 ± 0.000	0.772 ± 0.011	0.142 ± 0.001
			10607bF	0.305 ± 0.001	0.717 ± 0.004	0.202 ± 0.004	0.992 ± 0.004	0.173 ± 0.001
			10607bH*	0.255 ± 0.001	0.612 ± 0.001	0.176 ± 0.002	0.736 ± 0.008	0.135 ± 0.000
11912	724/15	4b and 4b/4c	10608bF	0.306 ± 0.000	0.696 ± 0.000	0.162 ± 0.002	0.983 ± 0.004	0.177 ± 0.006
			10608bH*	0.236 ± 0.001	0.587 ± 0.002	0.140 ± 0.002	0.622 ± 0.003	0.152 ± 0.005
			10609bF	0.294 ± 0.002	0.700 ± 0.001	0.190 ± 0.003	0.982 ± 0.007	0.165 ± 0.001
			10609bH*	0.249 ± 0.001	0.616 ± 0.001	0.168 ± 0.002	0.759 ± 0.000	0.131 ± 0.000

**Fig. 3.**
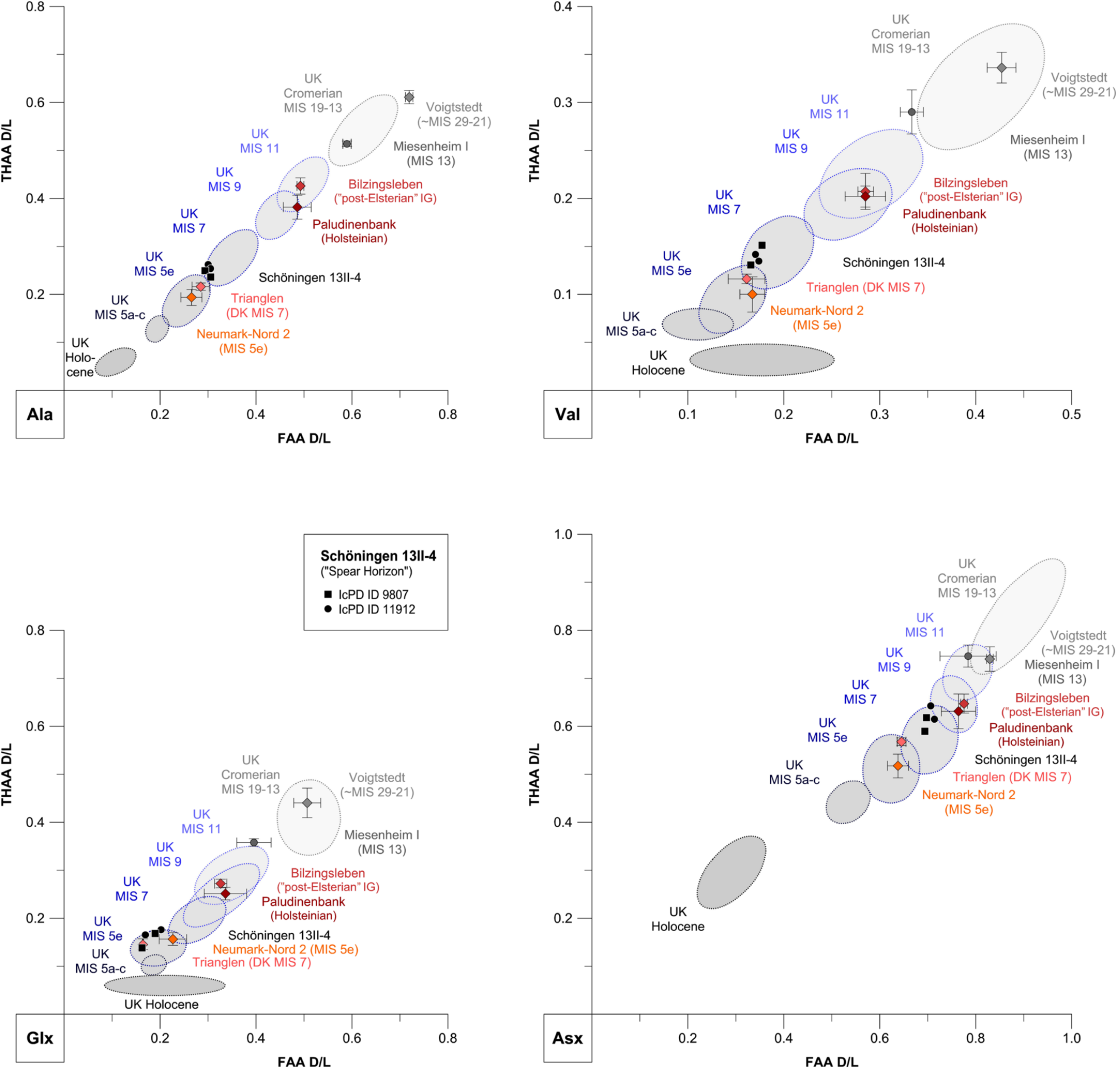
Results of IcPD analysis on *Bithynia* opercula. Free (FAA) versus total (THAA) D/L values of alanine (Ala), valine (Val), glutamic acid/glutamine (Glx), and aspartic acid/asparagine (Asx) from Schöningen 13II-4 bleached *B. tentaculata* opercula (Table 2) compared with shells from German and Danish sites: Voigtstedt (Artern Interglacial, ~MIS 29-21) (*84*); data from the latest Cromerian, pre-Elsterian site of Miesenheim I (MIS 13) (*83*) (see table S6); the post-Elsterian (MIS 11) site of Bilzingsleben (see table S6); the Holsteinian (MIS 11) Paludinenbank deposits in Berlin (see table S6); Copenhagen Trianglen (MIS 7) (*37*); and Neumark-Nord 2 (MIS 5e) (*38*, *39*). Schöningen and comparator sites lie between 50.4°N and 52.5°N latitude, except for Trianglen, which is located further to the north at 55.7°N latitude. While the integrated temperature histories will be slightly different, the transparent blue ellipses show the range of values exhibited by opercula from British sites (*35*) with age correlation to the MIS record (*78*). Note the differently scaled axes for each graph; each amino acid racemizes at a different rate, providing different levels of temporal resolution.

Measured against an AAG framework established for *Bithynia* opercula on the British Isles (*35*), the Schöningen 13II-4 Spear Horizon opercula dataset clearly falls within the younger range of sites attributed to MIS 7 (Fig. 3 and Table 2). However, the same integrated temperature history cannot be assumed for different regions (*36*), so comparison with geographically closer sites is crucial. Comprehensive opercula aminostratigraphies for continental Europe are now under development, and comparisons with initial data can already be made.

The Spear Horizon opercula D/L values (ratio of right- to left-handed amino acids; see Materials and Methods) are similar to those from the MIS 7 interglacial locality at Trianglen in Copenhagen, Denmark (*37*). Because Trianglen (~450 km to the north-northeast) likely experienced slightly colder integrated temperatures than Schöningen throughout the late Middle Pleistocene, D/L values from Trianglen would be slightly lower than at Schöningen for samples of a similar age. Compared with the last interglacial Eemian deposits (MIS 5e) of the nearby site of Neumark-Nord 2, Germany (*38*, *39*), the Spear Horizon opercula show slightly higher D/L values in the faster-racemizing alanine (Ala) and aspargine/aspartic acid (Asx), the two amino acids that provide the best temporal resolution for sites of this age (Fig. 3 and Table 2). Thus, the Spear Horizon is older than the Eemian deposits at Neumark-Nord 2. As the Cycle II sequence at Schöningen is overlain by a glacial till attributed to the Drenthian stage of the Saalian glaciation (Table 1), the Spear Horizon must also pre-date MIS 6. Overlapping D/L values of the slower-racemizing valine (Val) and glutamine/glutamic acid (Glx) from the Spear Horizon and from Neumark-Nord 2 (Fig. 3 and Table 2) indicate that the Schöningen samples cannot pre-date the Eemian Interglacial (MIS 5e) by several climatic cycles. Conversely, observed levels of IcPD in the Spear Horizon opercula dataset are substantially lower, and therefore younger, than those of the Holsteinian Paludinenbank deposits in Berlin (*40*) and the “post-Elsterian” interglacial fluvial deposits at the bottom of the Bilzingsleben (Germany) travertine attributed to MIS 11 (*41*). Considering that these comparator sites have experienced a similar temperature history, an age of MIS 7 is indicated for the Schöningen 13II-4 Spear Horizon, which is substantially younger than all earlier estimates (*1*, *30*–*32*).

These closed-system AAG opercula data are supported by low D/L values and age calculations for ostracod and horse teeth samples from the Schöningen 13II-4 Spear Horizon (see tables S4 and S5) that place the site near the end of the late Middle Pleistocene, although we concede that the age calculations derived from ostracod and horse teeth are still experimental and lack a robust comparator dataset. Stratigraphically, the Spear Horizon must pre-date the MIS 6 ice advance of the Drenthian (*13*, *14*, *19*–*21*), and all data from opercula, ostracod, and horse teeth samples agree with this chronology.

## DISCUSSION

### Chronostratigraphic implications

**Fig. 4.**
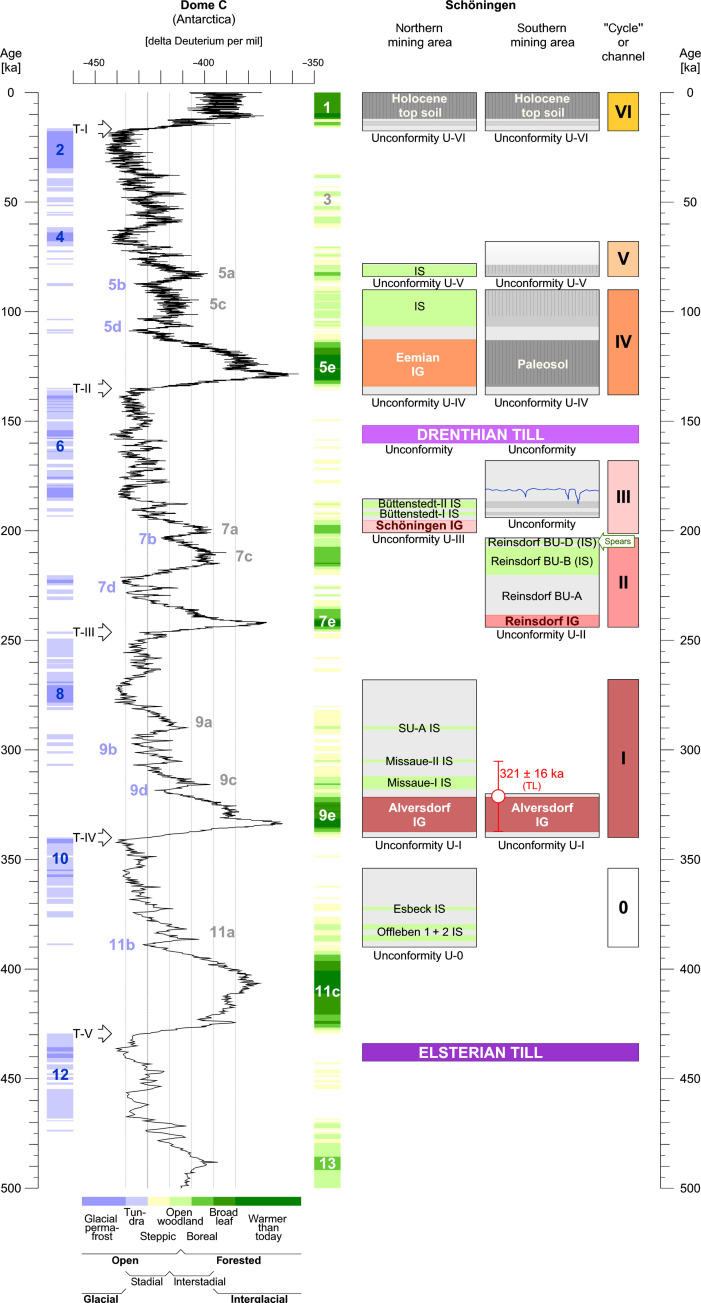
Major stratigraphic units of the Schöningen Pleistocene sequences. Schöningen stratigraphy is correlated with global MISs and the deuterium record of temperature change documented in the Antarctic Dome C ice core (*106*) over the past 500 kyr (see also table S2). Green arrow in the right column indicates the stratigraphic position of the Schöningen 13 II-4 Spear Horizon with the approximate age of ~200 ka. Colors of Cycles I to VI correspond to the color coding in figs. S1 to S3. Data from Schöningen northern mining area modified from (*12*, *25*); data from Schöningen southern mining area modified from (*13*, *26*); TL age estimates for Schöningen 13I-1 from (*31*). Color coding of ice core phases and MIS follows an extrapolation of environmental conditions in northern central Europe (*42*). T-I to T-V marks at the left denote the terminations of major glacial cycles. IG, interglacial; IS, interstadial; U, major unconformities.

The AAG data bring about a series of implications for understanding the late Middle Pleistocene chronostratigraphy at Schöningen and for establishing a more precise age for the 13II-4 Spear Horizon. A MIS 7 age pushes the archaeology of the Spear Horizon to a date roughly 100 kyr younger than previously considered. Consequently, this implies that channel formation and cycles of deposition at Schöningen would not in every case correspond to full interglacial-glacial cycles, where each started and ended with a major glacial termination. Instead, we propose a revised Schöningen chronostratigraphic model where correlations of the Schöningen Cycles with isotope chronologies are considered at substage levels, and Cycles II and III are both placed into MIS 7, with Cycle III also extending into early MIS 6 (Fig. 4; see also table S2).

With Cycle II dating to MIS 7, the preceding Cycle I Alversdorf Interglacial can be placed into the peak phase of MIS 9 (i.e., MIS 9e), which is in full agreement with the TL age established for the Schöningen 13I-1 site (Fig. 4) (*31*). However, this contradicts earlier age models that correlate the Cycle I Alversdorf deposits to the Holsteinian of MIS 11. The basic argument for this correlation was that Cycle I interglacial deposits represent the first period characterized by fully interglacial conditions following the Elsterian glacial series at Schöningen. On the basis of its palynology, the Alversdorf Interglacial was argued to be similar to the Holsteinian (*25*), but its slightly different pollen successions suggested that it could represent a local variant of the Holsteinian (*26*). Given the overall similarities between Hoxnian, Holsteinian, and Masovian Interglacial archives (*23*), such local variation of the Holsteinian at Schöningen is unlikely. Rather, the Alversdorf Interglacial represents an interglacial different from and younger than the Holsteinian sensu stricto (see table S3).

In northern central Europe, forested interstadials, like the Cycle 0 Offleben I, Offleben II, and Esbeck Interstadials that preceded the Alversdorf Interglacial deposits in the northern mining area of Schöningen (*25*), typically correlate to the later substages of the longer temperate periods [e.g., MIS 5a and 5c in Upper Pleistocene record (*42*)]. Interstadials equivalent to the Offleben I, Offleben II, and Esbeck are not documented elsewhere from late Elsterian deposits (*43*). On the contrary, whenever stratigraphic data are available, the Holsteinian succession lies immediately and concordantly on top of the Elsterian glacial series (*18*, *19*, *44*, *45*), indicating the rapid change from fully glacial to fully interglacial conditions, leaving no time for the establishment of interstadial forested environments (Fig. 4). Our revised age model (see table S2) not only reaffirms the MIS 9e age for the Alversdorf Interglacial (Cycle I) but also implies that no distinctly Holsteinian (MIS 11 peak = MIS 11c) deposits have yet been identified at Schöningen. As a result, the Offleben I, Offleben II, and Esbeck Interstadials would correspond to the later warming phases within MIS 11 (i.e., MIS 11a) rather than representing late MIS 12 warming intervals (*25*). The Cycle I Alversdorf Interglacial of MIS 9e was then trailed by further mild interstadial phases (Missaue I, Missaue II, and SU A), likely correlating with MIS 9c–a (Fig. 4).

Moving through the stratigraphic sequence, the Cycle II Reinsdorf Interglacial peak deposits would correlate to MIS 7e and the 13II-4 Spear Horizon to a mild interval toward the end of MIS 7. This is not contradicted by OSL data for layer 13II-2c1 that have been explicitly described as maximum age estimates (*32*), with single aliquots covering a wide range of individual measurements between ~500 and ~200 ka (see fig. S5).

The palynologically defined Schöningen Interglacial of the northern mining area succeeds Cycle II of the southern mining field and would correlate to the final phase of MIS 7 and the ensuing forested interstadials to the MIS 6 transition (Fig. 4; see also table S2). Cycle III deposits in the southern mining area do not preserve any sediments of clearly temperate character but frequently display cryogenic features, placing them partly into MIS 6 preceding the Drenthian ice advance.

In line with the regional chronology of the northern central European lowlands (see Supplementary Text), our data now provide four chronological reference points in the Schöningen sequence (Fig. 4 and see also table S2): (i) the Elsterian tills at the base of the sequence date to MIS 12 (*15*, *17*); (ii) the Alversdorf Interglacial represented at Schöningen 13I-1 dates to MIS 9 (*31*) but does not correspond to the Holsteinian sensu stricto (MIS 11); (iii) the amino acid data suggest a MIS 7 age for Schöningen 13II-4; and (iv) the late Saalian Drenthe tills belong to MIS 6 (*13*, *14*, *19*–*21*). With reference to the local biostratigraphic evidence (*27*), the Spear Horizon corresponds to the second forested interstadial phase (BU-D) after the Reinsdorf Interglacial peak, which likely correlates to a phase of climatic deterioration at the end of MIS 7c or within MIS 7b (Fig. 4; see also Supplementary Text, fig. S6, and table S2). In consequence, we suggest a late MIS 7 age around ~200 ka for the Spear Horizon and the world’s oldest complete hunting weaponry, roughly 100 ka younger than previously assumed.

With these chronostratigraphic implications, the four temporal reference points in the Schöningen sequence and revised age for the Spear Horizon can serve as the working foundations for a reevaluation of Middle Pleistocene mammalian biostratigraphy across short- and long-term climate cycles.

### Hominin behavioral implications

The Schöningen spears will be celebrated forever as spectacles of Paleolithic technology, yet their importance for understanding hominin behavioral evolution can only be fully appreciated within the wider context of the late Middle Pleistocene archaeological record.

The small lithic assemblage made of local flint underscores the site’s functional character and ephemeral use of raw materials in the Spear Horizon. Blank production, or any form of primary lithic reduction, played a minor role at the site. Evidence for the application of Levallois flaking strategies is absent. Instead, the assemblage consists largely of finished tools brought to the site and an abundance of small flakes, chips, and resharpening debris, with use wear hinting at activities related to butchery, woodworking, and hide scraping (*3*). Overall, the lithic assemblage is not indicative of any particular time period but is often attributed the Lower Paleolithic owing to the absence of prepared-core technology and the original older age estimates for the site (*3*).

Lacking direct dates for the Spear Horizon, there had long been a certain air of uncertainty surrounding the deep age of the site. With claimed ages of ~400 or ~300 ka for the Spear Horizon, Schöningen stood as an outlier among sites attributed to the Lower Paleolithic and to *Homo heidelbergensis* (*46*), the probable last common ancestor of early modern humans that evolved in Africa and the Neanderthals of western Eurasia (*47*). Our dating evidence for the Spear Horizon corrects this mismatch and aligns the Schöningen spears within the timeframe of European Neanderthals and the Middle Paleolithic. A late MIS 7 age does not diminish the significance of the site—the Spear Horizon archaeological record and associated behaviors stand unchanged—rather our revised age assignment positions the Schöningen findings in line with other evidence from this period of emerging Neanderthal behavioral complexity (*48*, *49*).

The Spear Horizon provides unequivocal evidence for the recurrent use of the Schöningen lakeshore as an ambush hunting ground at which entire horse family groups were targeted, killed, and butchered (*4*–*9*). This scenario of repeated hunting events generated a zooarchaeological signature similar to many Middle Paleolithic sites with large, mono-specific faunal assemblages attributed to Neanderthal hunting (*50*–*52*). Whereas the Schöningen hominins specialized in hunting horses, other sites show an equally strong preference for a single prey taxon, including aurochs at Biache-Saint-Vaast (France; MIS 7) (*53*), red deer at Grotte du Lazaret (France; MIS 6) (*54*), rhinoceros at Taubach (Germany; MIS 5e) (*55*), bison at Mauran (France; MIS 5) (*56*), horse at Zwoleń (Poland; MIS 5a/4) (*57*), and reindeer at Salzgitter Lebenstedt (Germany; MIS 5/4) (*58*, *59*), to mention only a few. By MIS 5e, Neanderthals regularly took down even the largest prey animals, as evidenced by the numerous butchered elephant carcasses from Neumark-Nord 1 (Germany) (*60*). Occasionally, mono-specific hunting has been interpreted from Lower Paleolithic sites, most notably reindeer in level L at La Caune de l’Arago (Tautavel, France) (*61*) and bison in TD 10.2 at Gran Dolina (Atapuerca, Spain) (*62*). However, the taphonomic histories of these cave sites are far more complicated, blurring interpretations of any discrete hunting events at these localities. Given this, it appears that mono-specific hunting coalesced into regular practice during MIS 7 and became widespread in later Middle Paleolithic contexts (*63*). To achieve this level of routine success, hunting forays at Schöningen and other mono-specific sites must have been wholly collaborative and group-minded efforts motivated by a communal set of objectives (*64*, *65*). The growing body of evidence for high levels of hunting collaboration implies that such Neanderthal social environments became a powerful evolutionary force during MIS 7, allowing for greater control over their environments and ultimately leading to increased impacts on the ecosystem by the late Pleistocene. The age estimate of ~200 ka realigns the Schöningen 13II-4 Spear Horizon with this formative period of Neanderthal hunting specialization.

## MATERIALS AND METHODS

### Amino acid geochronology

AAG relies on the time-dependent degradation of proteins within fossils. There are 20 naturally occurring amino acids in living organisms, and all but one have a chiral center, meaning that amino acids can exist in two chemically identical mirror image forms (enantiomers), like left and right hands. In living organisms, proteins are almost exclusively made from the left-handed form. After death, or upon cessation of tissue turnover, a spontaneous racemization reaction occurs gradually to redress the imbalance until there is a racemic equilibrium of left- (L) and right-handed (D) amino acids. The proportion of D-to-L amino acids is therefore a measure of the extent of protein degradation and, if this is predictable over time (e.g., within a closed system), can be used to estimate the age of a sample (*66*, *67*).

### Sample collection

Opercula (*B. tentaculata*) and ostracod (*Herpetocypris reptans* and *Prionocypris zenkeri*) samples were collected from sediment blocks lifted from the original excavation conducted by Hartmut Thieme and the Niedersächsisches Landesamt für Denkmalpflege (NLD) between 1992 and 2008 (Fig. 2; see Supplementary Text and fig. S4) (*11*, *68*). These blocks were initially intended to preserve important or fragile faunal specimens and now represent the last remaining sedimentary record from the main find concentration of the Spear Horizon. Over the course of the excavation, nearly 50 sediment blocks were transported to the NORDFROST GmbH & Co industrial freezer facility near Barsinghausen, Germany and stored under subzero (degrees Celsius) temperature until processing began in 2014 at the NLD and later at the Paläon Forschungs-und Erlebniszentrum Schöninger Speere (Forschungsmuseum Schöningen) in Schöningen, Germany. The blocks were thawed slowly under controlled conditions inside a laboratory fume hood to prohibit mold growth. After consolidating and extracting the bones of interest, half of the remaining portion of each block was excavated, and the sediment was water-sieved by layer through nested screens; sediment columns were also prepared for micromorphology and pollen analyses (*69*).

Samples from horse (*Equus mosbachensis*) permanent molars originated from the 1992–2008 NLD excavations at Schöningen 13II-4 (Fig. 2; see also Supplementary Text) (*11*, *68*). Dentine powder was extracted from the buccal surfaces of the horse teeth by diamond-headed drill bit at the Curt-Engelhorn-Zentrum Archäometrie gGmbH in Mannheim, Germany.

In total, four opercula, 15 ostracod valves, and nine dentine collagen samples from eight horse molars were subjected to AAG analysis (Fig. 2). All samples originated from the Schöningen 13II-4 Spear Horizon, layers 4b, 4b/4c, and 4c.

### Opercula analysis

Analysis of *B. tentaculata* opercula samples combined the isolation of an “intracrystalline” fraction of amino acids by bleach treatment (*70*–*73*) with RP-HPLC (*74*), performed at the North East Amino Acid Racemization Laboratory, University of York, UK. This combination of techniques generates D/L values of amino acids from the chemically protected protein within the biomineral, thereby enabling decreased sample sizes and increased analytical reliability.

For pretreatment, individual opercula were sonicated, rinsed multiple times in HPLC-grade water, and air-dried. To isolate the intracrystalline fraction, each cleaned operculum was transferred to a plastic microcentrifuge tube and crushed, and 50 μl of 12% NaOCl was added per milligram of the powdered sample. The tubes were shaken, rested for 24 hours, reshaken, and soaked again for 24 hours, and the NaOCl was pipetted off. The remaining powder was rinsed with H_2_O, centrifuged, and rinsed again; this process was repeated five times. For complete removal of the bleach, HPLC-grade methanol was added, left to rest for a few minutes, centrifuged, and pipetted off, and the bleached powder was air dried overnight. The bleached, dry powder was split into two subsamples: one for the analysis of the free amino acid (FAA) fraction and one for the total hydrolysable amino acid fraction (THAA). The FAA subsamples were demineralized with 10 μl of 2 M HCl per milligram of CaCO_3_ and dried overnight in a centrifugal evaporator; the THAA subsamples were demineralized in 20 μl of 7 M HCl per milligram of CaCO_3_. The subsamples were sealed under N_2_ and hydrolyzed by heating at 110°C for 24 hours. Following hydrolysis, vials were placed in a centrifugal evaporator to dry overnight.

Samples were rehydrated with 0.01 mM HCl and l-*homo*-arginine (internal standard synthetic amino acid) and analyzed in duplicate by RP-HPLC using fluorescence detection (*75*). A sample solution volume of 2 μl was mixed online with 2.2 μl of derivatizing reagent (260 mM *N*-isobutyryl-l-cysteine and 170 mM *o*-phthaldialdehyde dissolved in 1.0 M potassium borate buffer, adjusted to pH 10.4 with KOH) immediately before injection. Derivatized amino acids were separated with C18 HyperSil BDS C18 reverse-phase columns (5 μm; 250 mm length by 4 mm diameter) at 25°C using a linear gradient elution of sodium acetate buffer (eluent A; 23 mM sodium acetate trihydrate with 1.5 mM sodium azide and 1.3 μM EDTA, adjusted to pH 6.00 ± 0.01 with 10% acetic acid and 10 M sodium hydroxide), HPLC-grade methanol (eluent B), and HPLC-grade acetonitrile (eluent C).

During preparative hydrolysis, both asparagine and glutamine undergo rapid and irreversible deamination to aspartic acid (Asp) and glutamic acid (Glu), respectively (*76*). It is therefore impossible to differentiate these amino acids from their derivatives, and they are reported together as Asx and Glx. The D/L values of aspartic acid/asparagine (Asx), glutamic acid/glutamine (Glx), serine (Ser), alanine (Ala), and valine (Val), as well as the (Ser)/(Ala) value, provide reliable estimates of protein decomposition. Serine is geochemically unstable, with one of its decomposition products being alanine (*77*). This enables the ratio of the concentration of serine (Ser) to the concentration of alanine (Ala) to be used as a useful indication of the extent of protein decomposition. The D/L value of an amino acid will increase with increasing time, while the (Ser)/(Ala) value will decrease. Each amino acid racemizes at different rates and therefore is useful over different timescales. The D/L of serine is less useful as a geochronological tool for samples of this age, but aberrant values may indicate contamination. In a closed system, the amino acid ratios of the FAA and the THAA subsamples should be highly correlated, enabling the recognition of compromised samples (*35*, *78*, *79*). Amino acid data from the Schöningen opercula are consistent with closed-system proteins and therefore can be reliably used for age estimation.

The extent of IcPD in both the FAA and THAA increases with time, with increased levels of protein breakdown during warm climatic stages and a slowing in the rates of degradation in cold stages. Over a small geographical area, it can be assumed that the integrated temperature histories are effectively the same. Given a similar temperature history, this then allows an aminostratigraphic framework for an area to be developed, plotting the FAA against the THAA data, with younger samples plotting toward the bottom left-hand corner of the graph and older samples toward the top right-hand corner of the graph. In this sense, IcPD represents a relative chronology, notwithstanding a regional thermal gradient (*80*). Independent geochronology allows these clusters to be correlated to the marine oxygen isotope record; such a framework has been developed for opercula from the United Kingdom (*35*, *78*). The Schöningen samples have amino acid ratios that are slightly higher than those for the same species from sites correlated with the British Ipswichian and therefore the Eemian and MIS 5e (Fig. 3 and Table 2) (*81*). However, a direct correlation must be taken with caution owing to the likely differences between the temperature histories of continental and British Pleistocene sites. To address this, a comparison was made between the Schöningen samples and an initial dataset of other German and Danish sites with more comparable temperature histories: the MIS 5e site of Neumark-Nord 2 (*38*, *39*, *82*); the independently dated MIS 7 site at Copenhagen Trianglen (*37*); IcPD data from the Holsteinian Paludinenbank layer in Berlin (MIS 11) (see table S6); the post-Elsterian (MIS 11) interglacial gravels at the base of the Bilzingsleben travertine (see table S6); the latest Cromerian, “pre-Elsterian” site of Miesenheim I [MIS 13 (*83*)] (see table S6); and the Artern Interglacial site of Voigtstedt [~MIS 29-21 (*84*)].

### Ostracod and tooth analysis

Ostracod valves were removed from the sediment samples with a needle, cleaned ultrasonically in distilled deionized (DDI) water, and rinsed with DDI water to remove sediment. Some valves were additionally cleaned with a small brush under a binocular microscope to eliminate fine debris. The cleaned valves were subsequently submerged in 3% H_2_O_2_ for 2 hours to remove secondary organic molecules adsorbed to the shells (*74*, *85*). Only translucent specimens were selected for analysis, as these are often better preserved and as a potential means for excluding reworked samples. Dentine powder was extracted from a hole (2 mm in diameter, 5 to 10 cm in depth) bored into the buccal surfaces of horse teeth with a diamond-headed drill bit. After demineralization, the dentine samples were purified and concentrated by dialysis (3.5 kDa) (*86*, *87*). All ostracod and dentine samples were prepared and subjected to amino acid analysis using an RP-HPLC (*75*, *85*) in the Biomolecular Stratigraphy Laboratory (Laboratorio de Estratigrafía Biomolecular) at the Universidad Politécnica de Madrid, Spain.

The samples were sealed under N_2_ in 20 μl of 7 M HCl per milligram of dentine or 7 μl of 6 M HCl per milligram of ostracod and hydrolyzed by heating at 100°C for 20 hours to release peptide-bound amino acids. The hydrolysates were evaporated in vacuo, rehydrated in 7 μl of 0.01 M HCl with 1.5 mM sodium azide and 0.03 mM l-*homo*-arginine (internal standard synthetic amino acid), and analyzed using an Agilent-1100 HPLC equipped with a fluorescence detector. Excitation and emission wavelengths were programmed at 230 and 445 nm, respectively. Derivatization took place before injection by mixing 2 μl of sample solution with 2.2 μl of precolumn derivatization reagent, consisting of 260 mM isobutyryl-l-cysteine chiral thiol and 170 mM *o*-phthaldialdehyde dissolved in 1.0 M potassium borate buffer solution at pH 10.4. Derivatized amino acids were separated with Hypersil BDS C18 reverse-phase columns (5 μm; 250 mm length by 4 mm inner diameter) using a three-solvent linear gradient elution of sodium acetate buffer (eluent A: 23 mM sodium acetate trihydrate with 1.5 mM sodium azide and 1.3 μM EDTA, adjusted to pH 6.00 with 10% acetic acid and 10 M sodium hydroxide), HPLC-grade methanol (eluent B), and acetonitrile (eluent C). The linear gradient was performed at 25°C with a flow rate of 1.0 ml/min, from 95% eluent A and 5% eluent B upon injection to 76.6% eluent A, 23% eluent B, and 0.4% eluent C at 31 min.

For ostracods, the analysis of multiple amino acids yields largely redundant information on sample age (*88*); therefore, only the aspartic acid/asparagine (Asx) and glutamic acid/glutamine (Glx) constituents of the ostracod valves were analyzed, as Asx and Glx account for ~50% of the amino acid content in most ostracod valves (*85*, *89*, *90*). To establish the ages of the ostracod samples, D/L values of Asx and Glx were entered into the equations established for *Cyprideis torosa* and *H. reptans* from central and southern parts of the Iberian Peninsula (D/L Asx < 0.401 and D/L Glx < 0.140) (*91*)

$$\begin{array}{c} \sqrt{\text{Age}\;\left(\text{ka}\right)}=-3.586+19.745\text{Ln}\left(\frac{1+D/L\;\text{Asx}}{1-D/L\;\text{Asx}}\right);\\ r=0.993,\;P=0.001 \end{array}$$

$$\begin{array}{c} \sqrt{\text{Age}\;\left(\text{ka}\right)}=-3.186+58.972\text{Ln}\left(\frac{1+D/L\;\text{Glx}}{1-D/L\;\text{Glx}}\right);\\ r=0.989,\;P=0.001 \end{array}$$

Since *H. reptans* was included in the analyzed samples, the D/L values were directly comparable without a conversion factor; also, *C. torosa* and *H. reptans* show similar racemization rates (*91*). However, *P. zenkeri* analyzed from Schöningen may show some differences in racemization patterns when compared with those of *H. reptans*.

A cutoff value of 0.8 in the concentration of l-serine to that of l-aspartic acid was used to determine anomalous samples as serine decomposes rapidly (*92*), and excessive amounts can indicate contamination by modern amino acids. Two analytical samples (Schöningen ID 11965) suffered this kind of contamination and were rejected for the age calculation.

For the horse tooth samples, only aspartic acid (Asx; as due to the preparation method it is composed of both aspartic acid and asparagine) was used in the analysis as it racemizes faster and has been shown to provide better age estimates for tooth samples (*93*–*98*). In many cases, the D enantiomers of other amino acids could not be identified in the chromatograms or appeared in such low amounts that the D/L values were not suitable for differentiating between samples (e.g., leucine and isoleucine).

Numerical ages for the horse dentine samples were calculated from D/L values of aspartic acid (Asx) with the equation established for dentine collagen of mammals from the Iberian Peninsula (D/L Asx < 0.40) (*98*)

Age (ka)=−10.4+868.1*D/L Asx;r=0.97, P<0.001

Although amino acid racemization depends on the taxon considered (*99*), these algorithms were used on the assumption that collagen racemization rates are not affected by genus (*98*).

## Supplementary Materials

### This PDF file includes:

Supplementary Text Figs. S1 to S6 Tables S1 to S6 References
