# Supplementary material for: Revised age for Schöningen hunting spears indicates intensification of Neanderthal cooperative behavior around 200,000 years ago
Source: Sci Adv. 2025 May 9;11(19):eadv0752. doi: 10.1126/sciadv.adv0752 (PMC12063642; doi:10.1126/sciadv.adv0752)
Supplement: Supplementary file 1 — Supplementary Text Figs. S1 to S6 Tables S1 to S6 References [file sciadv.adv0752_sm.pdf]

Science Advances
AAAS

Supplementary Materials for

Revised age for Schöningen hunting spears indicates intensification of Neanderthal cooperative behavior around 200,000 years ago

Jarod M. Hutson *et al.*

Corresponding author: Jarod M. Hutson, jarod.hutson@leiza.de; Olaf Jöris, olaf.joeris@leiza.de

Sci. Adv. **11**, eadv0752 (2025)
DOI: 10.1126/sciadv.adv0752

This PDF file includes:

Supplementary Text
Figs. S1 to S6
Tables S1 to S6
References

Supplementary Text

Terrestrial Middle Pleistocene chronostratigraphy of northern central Europe

Since the Lower (~2,588–781 ka) to Middle Pleistocene (~781–126 ka) transition, global climatic oscillations are characterized and dominated by a ~100 ka cyclicity, as is documented in long and high-resolution isotope records (*106–108*). Almost all over the globe, these climatic fluctuations caused tremendous environmental changes. At northern mid-latitudes, long and pronounced glacial periods, during which inland ice sheets formed and open environments were established throughout periglacial zones, alternated with milder, often moister, and generally shorter periods when woodlands and soils developed (*22*). These mid-latitude temperate periods are defined as interglacials based on palynological criteria, when floral composition roughly compares with Holocene signatures and is characterized by thermophilous species. During some of the maximum cold stages, northern central Europe experienced the advance of inland ice masses from Fennoscandia. Three major advances can be traced over almost all of northern central Europe (Fig. 1 and Table 1): the last glaciation (Weichselian), the penultimate glaciation (Saalian, comprising an older Drenthe and a younger Warthe ice advance), and an earlier glaciation (Elsterian, comprising at least two ice advances) (*16*, *22*, *43*).

Across the northern European Lowlands, glacial geomorphodynamics led to major landscape remodeling. Evidence of earlier glaciations has been regularly removed or overprinted by later ice advances, resulting in mostly discontinuous terrestrial stratigraphies. Some older glacial deposits preserved only because the extent of younger inland ice advances was smaller than the preceding glaciation(s). Likewise, ice margins from the early phases of glacial build-up and advance are poorly known, since these were overrun during the maximum advances and covered with ground moraines across much of the northern European Lowlands. In contrast, maximal ice advances and stadial (cold) phases within periods of deglaciation left clear traces on the landscape, such as terminal moraines, deep tunnel valleys, and dead-ice topography. With the retreat and collapse of the ice sheets during periods of deglaciation, depressions and basins were left and quickly filled during the immediately succeeding interglacial. Outside formerly glaciated areas, interglacial deposits are rarely preserved in terrestrial environments, as interglacial environmental conditions at mid-latitudes predominantly led to weathering, soil formation, and landscape stabilization, rather than to sediment accumulation. Terrestrial interglacial deposits could form and preserve only in comparably small depressions, mostly in limnic environments but sometimes also in riparian channels. Outside formerly glaciated areas, depressions such as volcanic craters, tectonic or halokinetic basins, subrosion structures, and other such depressions are rare, resulting in fragmented sedimentary chronologies and limited regional comparability of interglacial archives.

Due to the fragmentary nature of terrestrial sequences, reliable linkages between global oxygen isotope stages (OIS) or other continuous paleoclimate records are often difficult to establish. The paleoclimate record that provides the most useful context is the marine oxygen isotope stage (MIS) record, in which odd-numbered stages refer to interglacials and even-numbered stages represent glacials (*107*, *108*). A further consequence of the discontinuous and patchy presence of temperate climate archives is that local site names are frequently used for interglacial or interstadial deposits, the latter representing temperate ameliorations within colder (stadial) periods that did not reach temperature levels as high as those of the interglacials.

Above all, an inordinately rigid distinction between glacial and interglacial phases within the MIS scheme oversimplifies the complex patterns of past climatic change documented in high-resolution marine and polar isotope records. Beyond the Pleistocene flickering between glacial and interglacial conditions, longer and shorter interstadials are well-documented in numerous paleoclimate archives. It is important to note that some interstadial phases may have reached levels close to those known from interglacial periods, albeit significantly cooler than the highest

interglacial temperatures. The palynological characteristics of the late Middle to Upper Pleistocene interglacials that can be most regularly traced over northern central Europe are summarized in table S3.

When considering the terrestrial record, only a few fixed points are accepted in the Pleistocene chronostratigraphy of northern central Europe: Weichselian (MIS 2) tills are directly overlain by Late Glacial and Holocene (MIS 1) sequences; Saalian (i.e., Drenthe and Warthe) tills, which date to MIS 6 (*20*), are overlain by palynologically-defined interglacial basin fills of the Eemian Interglacial (~MIS 5e) type (*16*, *22*, *43*); and Elsterian glacial sediments are overlain by interglacial deposits of the palynologically-defined Holstein Interglacial (*43*). In a core at Hamburg-Dockenhuden, Germany, a marine facies of Holsteinian sediments concordantly follows a sequence of clays (Lauenburger Ton/Lauenburg Clay) attributed to the late Elsterian (*19*, *44*), providing evidence for rapid sea level rise before isostatic uplift. This situation compares well to the Wacken outcrop where a marine boreo-arctic interglacial assigned to the Holsteinian is found atop red clays that follow a sequence of brown clays with arctic foraminifera attributed to the Elsterian. At the top of the Wacken locality (Schleswig-Holstein, Germany), Holsteinian deposits become increasingly brackish and contain a boreal to temperate fauna (*45*). In numerous other northern central European localities further inland, limnic Holsteinian deposits also overlay tills, sands, and clays assigned to the Elsterian. Given this, the glacial/interglacial coupling of the Elsterian/Holsteinian is regarded as well-established, and recent research has fixed the placement of the Elsterian glaciation and the succeeding Holsteinian within the MIS sequence (*15–18*), as is manifested in the correlation model of the Subcommission on Quaternary Stratigraphy of the International Commission on Stratigraphy (Elsterian = MIS 12; Holsteinian = MIS 11) (*18*).

The late Middle Pleistocene of Schöningen

Beginning in the 1980s, late Middle Pleistocene exposures were monitored and studied in the Schöningen open-cast lignite mine complex (*109*). Mining operations were formerly divided by a railway embankment ("Deutsche-Bahn-Pfeiler" or "DB-Pfeiler") into northern and southern fields. Large-scale work at the mine ceased in 2016. The Schöningen Middle Pleistocene stacked record (Fig. 4 and Table 1; see also table S2) combines stratigraphic and biostratigraphic observations from the northern and southern mining areas (*13*), together comprising several temperate phases of interglacial character between Elsterian and Drenthian (Saalian) tills.

Two Middle Pleistocene interglacials have been documented in the northern mining area: the locally-defined Alversdorf Interglacial, assumed to be equivalent to the Holsteinian, and the locally-defined Schöningen Interglacial (*12*, *25*). The southern mining area preserved a related sequence of interglacial occurrences between the two glacial series (Elsterian and Drenthian), with three cycles of lake level fluctuations (Cycles I–III, from bottom to top and old to young) marked by successions of fluvial (gravelly sands and sands), lacustrine (calcareous and organic muds, organic clays, silts, peats), and aeolian (silts, loess) deposits. These deposits were initially thought to represent successive interglacial-glacial cycles (*14*, *110*). According to palynological characteristics, the lacustrine/limnic/telmatic phases of the two older cycles are of temperate "interglacial" type, locally-defined as the Alversdorf Interglacial (Cycle I) and the Reinsdorf Interglacial (Cycle II). Deposits from the youngest cycle (Cycle III) are not explicitly temperate, but comprise silty deposits with paleosol formation, indicative of interstadial or interglacial conditions. Although no pollen spectrum is available for the Cycle III deposits in the southern mining area, the paleosols may be linked temporally with the palynologically defined Schöningen Interglacial of the northern mining area (*13*, *111*, *112*), as both are intersected by MIS 6 Drenthian (Saalian) glacial deposits. Importantly, at no single locality were pollen-containing sediments attributed to all of these three cycles observed in stratigraphic superposition, leading to skepticism towards the stratigraphic validity of the relative sequence (*21*) and its frequent re-interpretation (*32*) (Table 1; see also table S2). Nevertheless, the relative order of this sequence has been

documented by tracking intersections of the younger deposits into the older deposits across a series of stratigraphic profiles spanning from the northern to the southern mining fields (*14*) (see figs. S2 and S3).

Considering the northern and southern mining areas together, the Pleistocene sequence begins with moraines of the Elsterian glacial series. In the northern mining field, the succeeding Alversdorf Interglacial of Cycle I is preceded by a series of forested interstadials (Offleben I, Offleben II, and Esbeck) that have been assigned to a late Elsterian age (*12*). Here, these pre-Alversdorf deposits are referred to as "Cycle 0" (Figure 4; see also figs. S2 and S3, table S2). The Alversdorf interglacial at Schöningen has been ascribed to the Holsteinian as both revealed the first fully interglacial pollen signatures following the Elsterian (*12*, *25*) (Table 1; see also table S2). Until coring of the Ummendorfer Kessel in the nearby Aller Valley, the intermediate Reinsdorf Interglacial from the southern mining area had no other counterpart in other terrestrial late Middle Pleistocene records, but now can be correlated with the Horstwiesen Interglacial based on palynology (*113*) (see table S3). The Schöningen Interglacial of the northern mining area compares to other northern central European interglacial occurrences, regionally named Wacken, Dömnitz, or Leck Interglacials, based on palynological characteristics (*12*, *25*). Finally, the entire late Middle Pleistocene sequence at Schöningen is sealed under the ground moraines of the Drenthian advance of the late Saalian glaciation (*14*) (Fig. 4 and Table 1; see also table S2). Overall, the Schöningen stacked stratigraphic sequence is similar to that from Ummendorf, with three interglacials atop Elsterian and below Drenthian tills (*113*).

Beyond the three interglacials bracketed by Elsterian and Saalian tills, the Schöningen record documents a number of temperate interstadial phases. Within Cycles I and II at Schöningen, sediments with interstadial pollen compositions document shorter, lower-amplitude temperate periods that followed fully interglacial conditions. Paleoenvironmental reconstructions based on aquatic and terrestrial fossils from the Cycle II Reinsdorf sequence (*114*), however, indicate that lake formation and shoreline development were more complex than what could be explained by a simple cyclical sedimentation model (*27*–*29*).

Within the Cycle I sequence, the temperature peak of the Alversdorf Interglacial is followed by several milder interstadial phases, locally termed Missaue I and Missaue II interstadials and interstadial SU A (from bottom to top), with characteristics of boreal forest-type environments (*13*) (Fig. 4). These interstadials would likely correlate to an early glacial phase, while the Alversdorf Interglacial would correspond to the peak interglacial. The palynological sequence of Cycle II shows that the Schöningen 13 II-4 "Spear Horizon" also represents interstadial conditions, originally defined as the Reinsdorf-B interstadial (*13*), and later incorporated into biostratigraphic unit BU-D (*27*). The temperate, boreal Reinsdorf BU-D phase follows the earlier Reinsdorf BU-B (formerly Reinsdorf-A interstadial) of similar pollen composition, which stratigraphically overlays the peak Reinsdorf Interglacial (Fig. 4; see also table S2).

Thermoluminescence (TL) dates for heated flint recovered from Schöningen 13I-1 (Cycle I) produced a weighted mean age of 321 ± 16 ka (*31*), which allows for a nominal attribution to MIS 10 to MIS 8 at the 2σ level. This contradicts an attribution of the Cycle I Alversdorf Interglacial deposits to MIS 11. Alternatively, the TL data fit with MIS 9e (Fig. 4) when considering the available environmental data (*31*). As the sampled flints originated from a stratigraphic level below Schöningen 13II-4 (Cycle II), the TL data thus provide a maximum age of MIS 9 for the "Spear Horizon."

Attempts to directly date the Reinsdorf Cycle II peat deposits from Schöningen 13II-2 yielded a ^{230}Th/U age of 290 ± 5 ka (*33*), roughly concordant with a MIS 9 age, but this date was later withdrawn due to open-system behavior of the dated samples (*34*).

Optically stimulated luminescence (OSL) dating of glaciofluvial sediments from Schöningen site 12 (DB-Pfeiler; see fig. S1) all resulted in minimum age estimates due to saturating signal behaviour (*115*). Deposits on top of the Cycle II Reinsdorf sequence assigned to the Saalian

period produced minimum ages of >405 ± 33 ka and >356 ± 27 ka, inconsistent with the chronostratigraphy of the Schöningen sequence, and thus have been rejected (*115*). The sole remaining minimum age result from the top of these deposits (>194 ± 10 ka) indicate a pre-MIS 6 age (*115*), but is considered less informative here given the apparent difficulties of dating such sediments.

Other OSL dating approaches yielded age estimates for sediments from Schöningen 13II-2c1 suggesting a correlation with MIS 9 (*32*). This layer, stratigraphically located slightly above layer 13II-2c3, represents a cold interval (BU-A) between the full-interglacial Reinsdorf Interglacial deposits and the succeeding Reinsdorf BU-B and BU-D interstadial phases of the late Cycle II sequence. Age estimates for Schöningen 13II-2c1 resulted in a maximum age of ~300 ka (see fig. S5 and table S2), corresponding to MIS 9b based solely on the numeric date. However, Tucci et al. (*32*) correlate the Schöningen 13II-2c cold interval to MIS 9d, as this is the first stadial interval following the peak interglacial phase of MIS 9e. The succeeding BU-B and BU-D interstadials would thus correlate to MIS 9c and MIS 9a, respectively. Consequently, the Reinsdorf Interglacial deposits would correlate to MIS 9e.

The OSL dating results for layer 13II-2c1 are considered maximum age estimates because of unknown potential residuals associated with the protocols ($pIrIr_{290}$ and IR-RF) employed and the likely differential bleaching of the sediment sampled (*32*). No attempts for residual correction were made because of the unknown bleaching conditions (*32*), which may result in severe age overestimation for old samples after residual correction (*116*). Considering the 2σ ranges of confidence of these maximum OSL ages, one data set actually allows an attribution to MIS 7, while other 2σ ranges for other sets fall just short of including MIS 7 (see fig. S5). Residuals can amount to several tens of Gy (*117*), a presence of which would change these data by 5 ka for every 10 Gy residual towards a younger age. Considering the potential age overestimation due to unknown residuals and a slight age overestimation due to a dose rate that was not always water saturated, as was assumed for the calculations, the OSL data for layer 13II-2c1 do not contradict an attribution to MIS 7. The single aliquots measured indeed cover a wide range of individual results spanning between ~500 and ~200 ka (*32*), and therefore would be consistent with an MIS 7 age. In this scenario the OSL-dated cold interval could alternatively correspond to MIS 7d (see fig. S5).

The succeeding Schöningen Interglacial, lacking *Abies*, was likely cooler than the preceding interglacials and correlates with the Dömnitz/Wacken Interglacial (*12*). The Schöningen Interglacial ended abruptly and was followed by the local conifer-dominated Büddenstedt I and Büddenstedt II Interstadials. This sequence is capped by the Drenthian moraines of the late Saalian glaciation.

Refining the age of the "Spear Horizon" in MIS 7

To narrow the position of the "Spear Horizon" within the MIS 7 timeframe is difficult given the scarcity of comparable high-resolution pollen sequences in northern central Europe.

The Neualbenreuth Maar (northeastern Bavaria, Germany) resolves the MIS 7 sequence in some detail (*118*). Located about 370 km to the south-southeast, the Neualbenreuth Maar sequence documents comparably high pollen counts of thermophilic species for MIS 7e, 7c, and 7a, indicating near-interglacial conditions for each of these sub-stages (*118*). Global temperature data (*106*) also show that MIS 7a and 7c reached peak temperature levels only slightly lower than those of MIS 7e (Fig. 4). This was not the case in MIS 5 or MIS 9, when MIS 5a/9a and MIS 5c/9c temperatures were significantly lower than those of MIS 5e/9e (*106*).

Although the Neualbenreuth record currently includes relatively coarse sampling resolution, the MIS 7e–b sequence compares well with the Reinsdorf Cycle II sequence at Schöningen, covering the peak phase of the Reinsdorf Interglacial through a stadial cold phase (BU-A) and the succeeding BU-B and BU-D interstadial phases (Fig. 4; see also fig. S6 and table S2). If this

correlation is correct, the first Reinsdorf interstadial (BU-B) would correlate to MIS 7c, and the second interstadial (BU-D) that contains the Schöningen 13 II-4 "Spear Horizon," and which was characterized by partly forested interstadial conditions (*13*) lacking thermophilic species (see table S2), would date into a late phase of MIS 7c, MIS 7b, or initial MIS 7a. The fact that the second interstadial (BU-D) in the southern mining area is stratigraphically overlain by (pre-Eemian) Cycle III telmatic deposits (see fig. S3) would most likely place at least parts of Cycle III into MIS 7a. Based on stratigraphic observations comparing the northern and southern mining area sequences, the Schöningen Interglacial of the northern mining area most likely corresponds to Cycle III of the southern mining area (*12*), although this link has not been firmly established (*119*). Instead, the Schöningen Interglacial may overlap with the latest part of Cycle II in the southern mining area.

The Schöningen Interglacial sequence recorded in the northern mining area seems to follow Cycle II of the southern mining field. Although of interglacial character, it was cooler than the Alversdorf and Reinsdorf peak interglacial phases (*13*), ended abruptly, and was followed by the conifer-dominated Büttenstedt-I and Büttenstedt-II Interstadials. Capped by the late MIS 6 till of the Drenthian glacial advance, this temperate sequence should be equivalent with the latest part of MIS 7, most likely MIS 7a, including some transitional MIS 7–6 temperate oscillations (Fig. 4; see also table S2). With this stratigraphic evidence and the presence of thermophilous plant species in the Schöningen Interglacial deposits, it is not possible for the "Spear Horizon," with its clear open forest-steppe character (*13*, *32*), to align within the peak phase of the Schöningen Interglacial in the northern mining area. As a result, a late MIS 7c to 7b age is suggested for the "Spear Horizon," estimated at ~200 ka (Fig. 4; see also table S2).

<u>Limits to ostracod and horse tooth samples</u>

Three concerns may limit the fidelity of age calculations when applied to the Schöningen ostracod and dentine datasets (see tables S4 and S5). First, whether ostracod valves and dentine provide a closed-system for protein degradation has been questioned (*120*). The inter-crystalline (open-system) fraction of amino acids in ostracod valves has been shown to be removed relatively quickly (within ~1 ka) (*89*), leaving only the residual intra-crystalline (closed-system) fraction for age estimation; the same, however, has not been determined for dentine collagen, thus exposing the samples to open-system diagenetic effects. Second, the ostracod and dentine age calculation algorithms were constructed for regions with a current mean annual temperature (CMAT) of ca. 12–15 ºC, whereas CMAT for Schöningen is ca. 9 ºC. It has been observed that the racemization/epimerization rate doubles for every ca. 4 ºC increase (*121*). Differences in isoleucine D/L values of as much as 0.12 were attributed to a ca. 2 ºC CMAT change for last interglacial marine *Glycymeris* shells from the Mediterranean (*122*). Similarly, oolitic samples of the same age (MIS 5e) from the Bahamas show differences of 0.12 in D/L values that were attributed to a warming gradient of 2 ºC between localities (*123*). There are currently no conversion factors or alternative algorithms to account for temperature differences for ostracod and dentine samples from European continental sites. It is noteworthy, however, that ostracods originating from pluvial Lake Chewaucan-Ana River (Oregon, USA), dated at 201–187 ka (transition from MIS 7a to MIS 6) and with a current mean annual temperature of 9.5 ºC, showed Asx and Glx D/L values between 0.346–0.361 and 0.157–0.182, respectively (*89*) (see table S4). The Asx D/L values from Ana River are only slightly lower than those obtained from Schöningen ostracods. Thirdly, racemization is a temperature-dependent chemical process that slows or stops during periods of extreme cold and accelerates under warmer conditions (*124*, *125*) so a direct linear relationship is not valid. For example, low temperatures during the Last Glacial slowed the racemization of archaeological limpets from 27–18 cal. ka BP in northern Spain, after which they followed a similar rate to that of shells in younger levels (*124*). The Schöningen samples endured

multiple cold periods during which racemization was reduced or fully interrupted, the degree of which is currently immeasurable and cannot be factored into the age calculation algorithms.

Mean ages calculate to 127 ± 34 ka for ostracods (see table S4) and 155 ± 28 ka for horse dentine (see table S6). The calculated age approximations are significantly younger than ages previously postulated (*1*, *30–32*) and the MIS 7 estimate proposed here. Observing the abovementioned concerns, these AAR age estimates must be considered as minima for the formation of the "Spear Horizon."

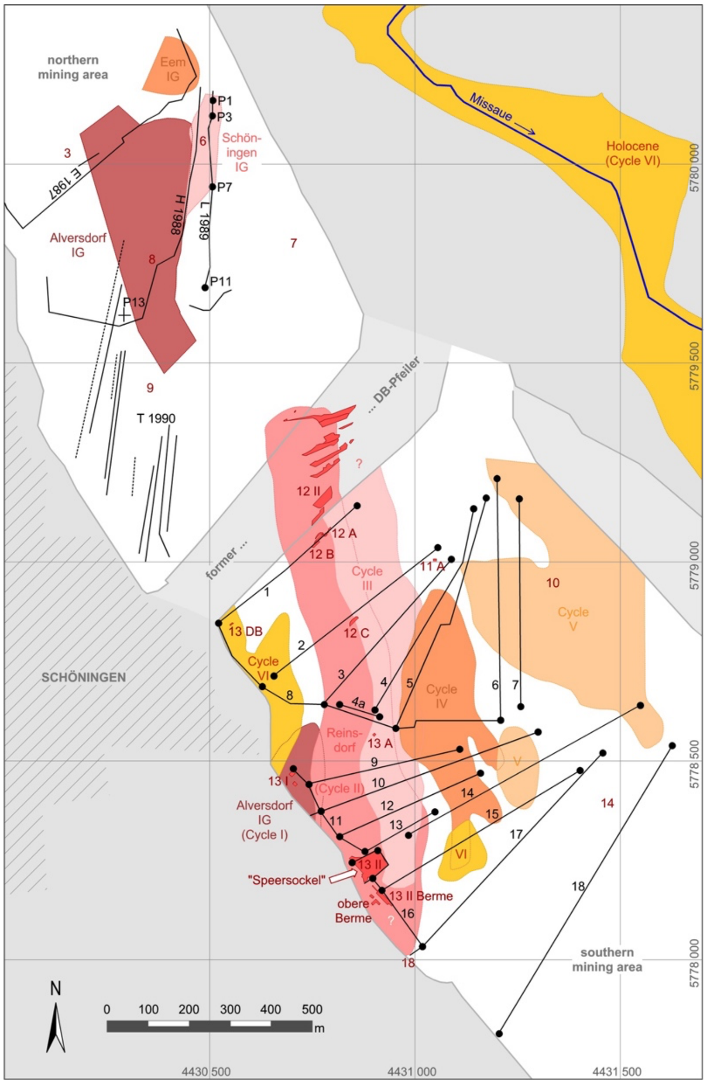

Fig. S1.

Map of the Schöningen mining complex and immediate vicinity. Map depicts deltaic-lacustrine deposits of the Alversdorf, Schöningen, and Eemian Interglacials in the northern mining area and the deposits of Cycle I (≈Alversdorf Interglacial), Cycle II (Reinsdorf sequence), and Cycle III (likely correlated to the Schöningen Interglacial of the northern mining area, or succeeding it) in the southern mining area. Cycles IV–VI cover the period from the Eemian to the Holocene (Table 1). Geological profile sections are denoted by numbered black lines; archaeological sites and excavation areas are shown in red. At least 20 Paleolithic archaeological sites have been identified within the mine complex (*11*), of which Schöningen 13II-4 "Spear Horizon" is the most well-known. The Schöningen 13II locality is situated along the western rim of the southern mining area in a location known as "Speersockel," which was preserved from destruction by bucket-wheel excavators in 1994. The nearby "Obere Berme" may represent a southern extension of the "Spear Horizon" from the main "Speersockel" excavation. Colors of Cycles I–VI correspond to the color-coding in Fig. 4 and figs. S2 and S3. Map compiled and modified from refs. (*14*, *32*, *109*, *119*).

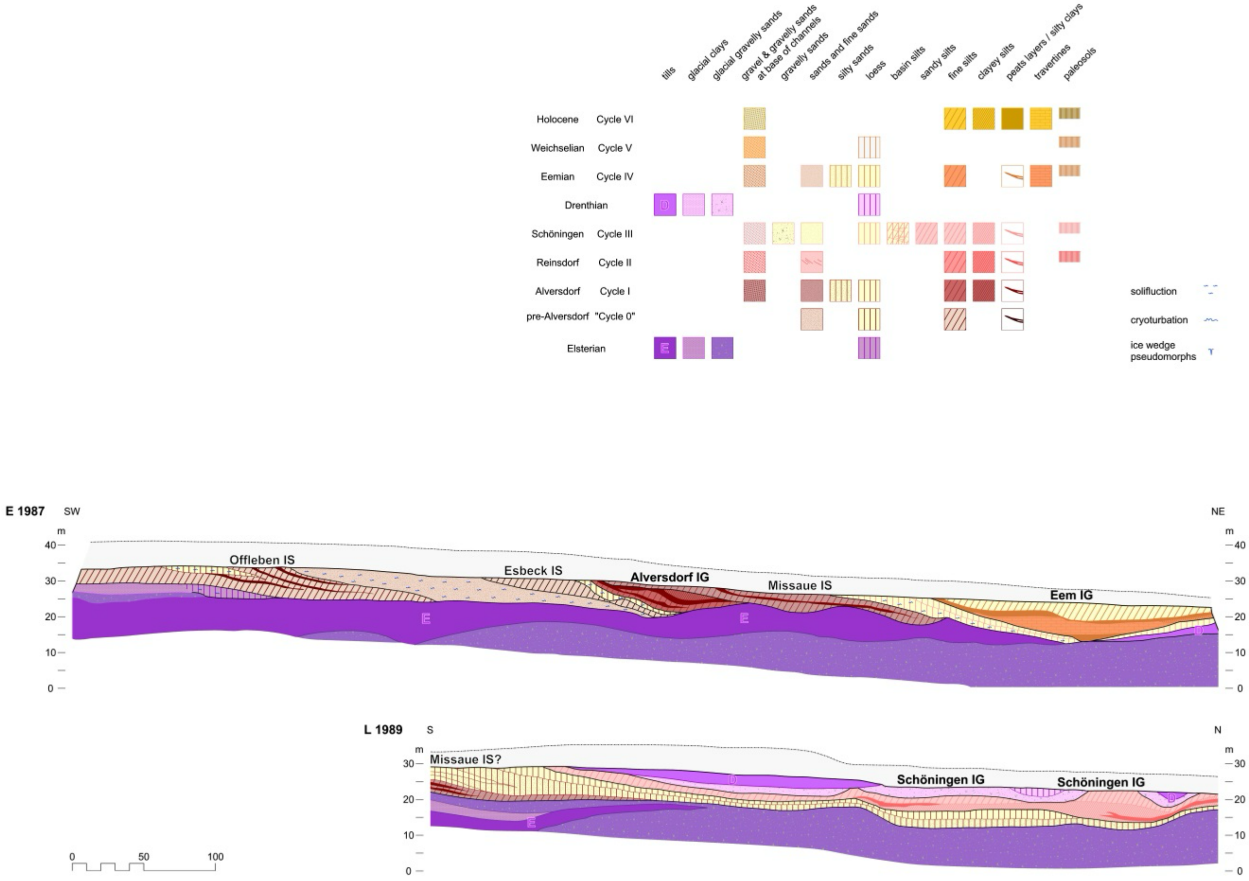

Fig. S2.
Selected profile sections from the Schöningen northern mining area. Profiles E 1987 and L 1989 show the deposits between the Elsterian (E) and Drenthian (D) glacial series. Profile sections modified from refs. (*14*, *25*). Colors correspond to the color-coding in Fig. 4 and fig. S1. Legend also applies to fig. S3.

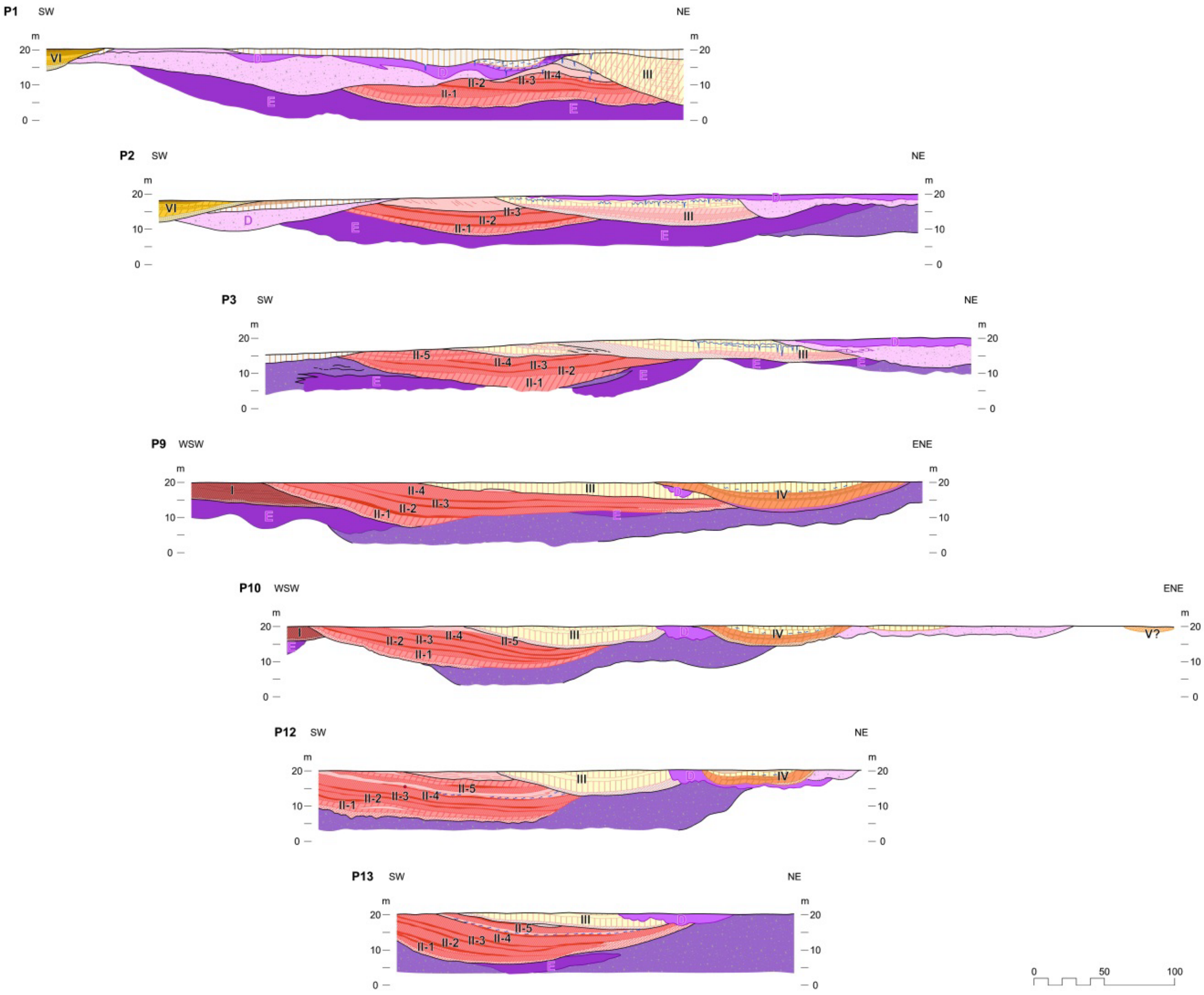

Fig. S3.
Selected profile sections from the Schöningen southern mining area. Profiles P1, P2, P3, P9, P10, P12, and P13show the deposits between the Elsterian (E) and Drenthian (D) glacial series and channel fills of Cycles I-VI. Profile sections modified from refs. (*14*, *25*). Colors of Cycles I–VI correspond to the color-coding in Fig. 4 and fig. S1. See fig. S2 for legend.

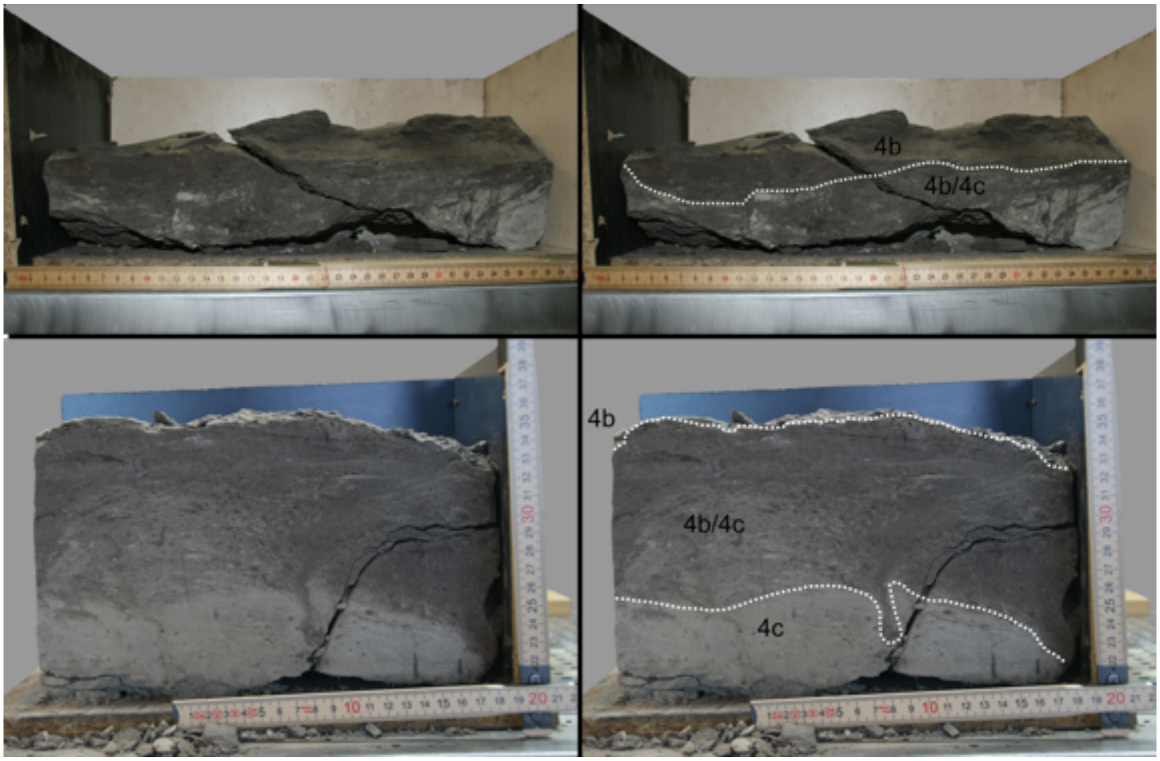

Fig. S4.
Schöningen 13II-4 sediment blocks. Two of the previously frozen sediment blocks preserving opercula samples submitted for amino acid racemization analysis: Schöningen sampling IDs 9807 (top images) and 11912 (bottom images). Block locations are shown in Fig. 2. All opercula samples came from the transition between layers 4b and 4b/4c, directly beneath the preserved faunal element resting atop each block.

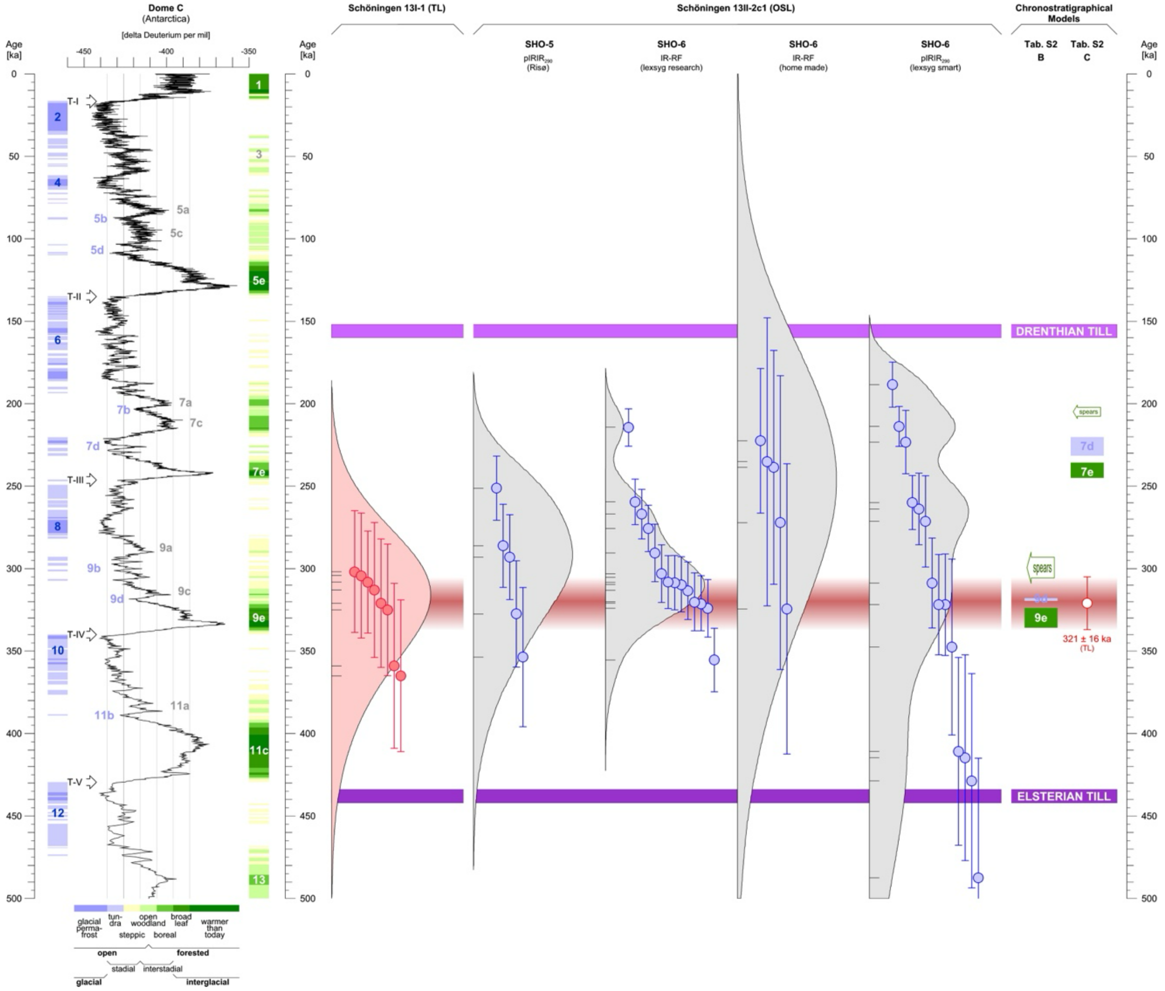

Fig. S5.

Dating results for Schöningen 13I-1 and 13II-2c1. Thermoluminescence (TL) dating results for site 13I-1 shown in red (*31*). Optically Stimulated Luminescence (OSL) dating results for samples unit 13II-2c1, samples SHO 5 and SHO 6, shown in blue (*32*). OSL samples were measured with different methods (pIRIR290 and IR-RF) and different readers (from left to right: Risø, lexsyg research, home made, lexsyg smart). Probability distribution curves are also shown, normalized by height. The stratigraphic position of the 13II-4 "Spear Horizon" above the dated level (13II-2) is shown to the right, between the Elsterian and Drenthian tills and against the mean value of 321 ± 16 ka for the TL-series of 13I-1 (*31*). Following the Schöningen standard chronostratigraphic model (see table S2), the "Spear Horizon" is correlated with late MIS 9, but conflicts with the TL age of 13I-1. According to the revised model presented here (see table S2), the "Spear Horizon" is placed into late MIS 7. Color-coding of ice core phases and MIS follows an extrapolation of environmental conditions in northern central Europe (*42*). IG = interglacial, IS = interstadial, T-I–IV marks at the left denote the terminations of major glacial cycles.

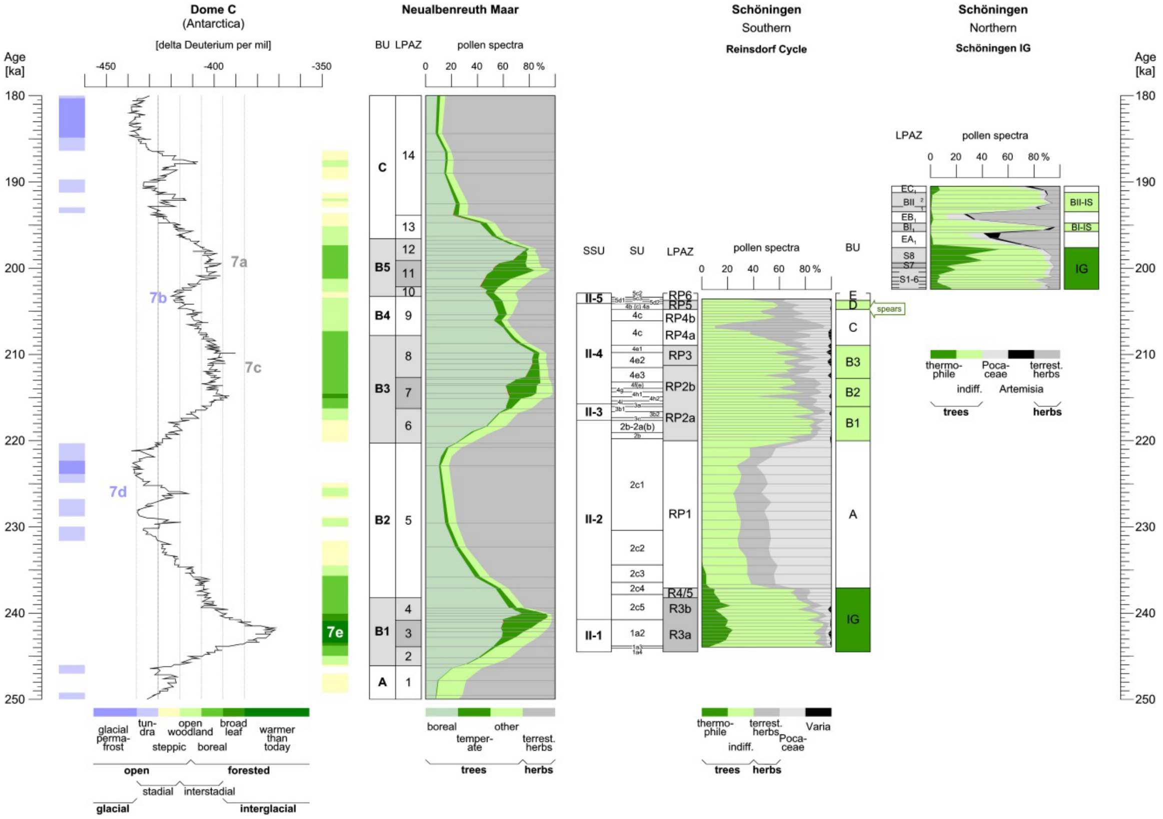

Fig. S6.

Marine oxygen isotope stage 7 pollen profiles from Schöningen and Neualbenreuth Maar. Tentative correlation of the Cycle II Reinsdorf sequence in Profile P13-II-2003 (*32*) of the southern mining area and the Schöningen IG sequence in Profile P7 (*13*) of the northern mining area with the Neualbenreuth Maar pollen sequence dated to MIS 7 (modified from ref. [118]) and the deuterium record of temperature change documented in the Antarctic Dome C ice core (*106*). For Schöningen, palynological phases of interglacial character are shown in dark green and those of interstadial character are shown in light green. LPAZ = Local Pollen Assemblage Zones; Neualbenreuth Maar; BU = biostratigraphic units; Schöningen BU = biostratigraphic units of the Reinsdorf sequence; Schöningen SSU = supraordinate sedimentary units; Schöningen SU = sedimentary units. Stratigraphic position of the Schöningen 13II-4 “Spear Horizon” at the base of LPAZ RP5/BU-D is indicated by a green arrow. Note uneven pollen sampling distances in the three records, indicated by horizontal grey lines in the pollen spectra.

Table S1.

Identified faunal taxa at Schöningen 13II-4. Number of identified specimens (NISP) and minimum number of individuals (MNI) per taxa from Schöningen 13II-4, including the "Spear Horizon." The complete faunal spectrum reflects full interglacial conditions, whereas the pollen spectrum from this layer indicates interstadial (open) woodland conditions (BU-D, formerly identified as the Reinsdorf-B Interstadial; see table S2). Note that unidentifiable specimens are not listed. (+) denotes taxon is present but not quantified.

Taxon	NISP	MNI
**Ungulate**		
Equus mosbachensis, Mosbach horse	9018	54
Equus hydruntinus, European ass	8	2
Megaloceros giganteus, Giant deer	7	2
Cervus elaphus, Red deer	358	7
Capreolus capreolus, Roe deer	7	1
Bos primigenius, Aurochs	79	4
Bison priscus, Steppe bison	59	2
Stephanorhinus hemitoechus, Narrow-nosed rhinoceros	1	1
Stephanorhinus kirchbergensis, Merck's rhinoceros	1	1
Palaeoloxodon antiquus, Straight-tusked elephant	4	1
**Carnivore**		
Panthera cf. *leo spelaea*, Cave lion	1	1
Canis lupus, Wolf	7	2
Vulpes vulpes, Red fox	3	1
Meles meles, European badger	5	1
Mustela sp., Weasel	5	1
**Small mammal**		
Castor fiber, Eurasian beaver	4	1
Talpa europea, European mole	3	1
Arvicola terrestris cantiana, European water vole	3	1
Microtus gregalis, Narrow-headed vole	1	1
Desmana sp., Desman	2	1
Sicista sp., Birch mouse	+	+
Chiroptera sp., Bat	1	1
**Bird**		
Anas acuta, Northern pintail	2	1
Anas crecca, Eurasian teal	4	1
Anas platyrhynchos, Mallard	3	1
Aythya fuligula, Tufted duck	2	1
Bucephala clangula, Common goldeneye	1	1
Cygnus olor, Mute swan	1	1
Rallus aquaticus, Water rail	1	1
Tadorna tadorna, Common shelduck	1	1
**Fish**		
Esox lucius, Pike	64	19
Cyprinidae sp., Carp/Minnow	3	1
Gasterosteus aculeatus, Three-spined stickleback	+	+
Perca fluviatilis, European perch	2	1
**Amphibian**		
Pelobates fuscus, Common spadefoot toad	1	1
Rana sp., Frog	4	1
Salamandridae sp., Salamander	+	+
**Insect**		
Coleoptera sp., Beetle	3	3

Table S2.

Late Middle Pleistocene deposits at Schöningen. The MIS and INQUA stratigraphic frameworks (*18*) are plotted against the Schöningen standard chronostratigraphic model (*13*, *27*–*29*) and the combined northern-southern mining field model presented here. IG = interglacial; IS = interstadial; BU = Biostratigraphic Units of the Cycle II Reinsdorf sequence (*27*); U – major unconformities; T-III–IV = terminations of major glacial cycles.

MIS	INQUA
**6**	**Saalian**
6/7a	
7a	
7b/7a	
7b	
7c	
7d	
**7e**	
T-III	
8	
9a/8	
9a	
9b	
9c	
9d	
**9e**	
T-IV	
10	
11a/10	
11a	
11b	
**11c**	**Holsteinian**
T-V	
late 12	
**12**	**Elsterian**

MIS	Schöningen standard chronostrati-graphic model	Cycle
6	**Drenthian**	
**7**	Büddenstedt-II IS<br>Büttenstedt-I IS<br>**Schöningen IG**	**IIII**
	unconformity	
8		
	Reinsdorf BU-E	**II**
**9**	Reinsdorf BU-D (IS)	
	Reinsdorf BU-C	
	Reinsdorf BU-B (IS)	
	Reinsdorf BU-A	
	**Reinsdorf IG**	
	unconformity	
10		**I**
11a/10		
**11**	SU-A IS<br>Missaue II IS<br>Missaue I IS<br>**Alversdorf IG**<br>**("Holsteinian")**	
	unconformity	
	Esbeck IS<br>Offleben I + II IS	
**12**	**Elsterian**	

MIS	Schöningen northern mining field	*Characterization*	Schöningen southern mining field	Cycle
**6**	Drenthian till	*ice advance*	Drenthian till	
6/7a	Büddenstedt-II IS<br>Büttenstedt-I IS	*interstadials (woodland phases)*	unconformity	**III**
7a	**Schöningen IG**	*interglacial*		
7b/7a	unconformity	unconformity	unconformity	U-III
7b		*(steppe-tundra)*	Reinsdorf BU-E	**II**
		interstadial (woodland phase)	Reinsdorf BU-D (IS)	
		(steppe - open woodland)	Reinsdorf BU-C	
7c		*interstadial (woodland and steppe woodland)*	Reinsdorf BU-B (IS)	
7d			Reinsdorf BU-A	
**7e**		*interglacial*	**Reinsdorf IG**	
T-III		unconformity	unconformity	U-II
8				**I**
9a/8	SU-A IS	*interstadial*		
9a	Missaue II IS	*interstadials (boreal-type flora)*		
9b				
9c	Missaue I IS	*interstadial (boreal-type flora)*		
9d				
**9e**	**Alversdorf IG**	*interglacial*	**Alversdorf IG**	
T-IV	unconformity	unconformity	unconformity	U-I
10				**0**
11a/10	Esbeck IS	*interstadial (boreal-type flora)*		
11a	Offleben I + II IS	*interstadials (boreal-type flora)*		
11b	unconformity	unconformity		U-0
**11c**				
T-V				
late 12				
**12**	Elsterian till	*ice advance*	Elsterian till	

Table S3.

Late Middle-to-Upper Pleistocene pollen sequence. Palynological characterization of late Middle-to-Upper Pleistocene interglacials of northern central Europe.

Interglacial	Characteristics	References
Eemian	Predominance of deciduous (thermophilous) trees, late spread of *Picea.*<br>Vegetational succession: *Betula-Pinus*, *Quercus*, *Corylus*, *Taxus*, *Carpinus*, *Picea*, *Abies* and a final *Pinus*-phase.<br>Absence of *Celtis*, *Pterocarya* and *Azolla*	*126–130*
Wacken / Dömnitz, Schöningen	Predominance of coniferous trees and *Alnus* (similar to Holsteinian), *Abies* partly lacking or in the late stage only (Dömnitz).<br>Vegetational succession: *Betula-Pinus* (*Juniperus*), *Alnus-Quercus* (*Pinus*), *Quercus-Corylus*, *Carpinus-Pinus*, *Carpinus-Picea* (*Abies*), *Pinus-Betula.*<br>Evidence of *Celtis* and *Azolla*; without *Pterocarya*	*131–136*
Reinsdorf / Horstwiesen	Similar to Holsteinian (predominance of coniferous trees and *Alnus*).<br>Vegetational succession: *Betula-Pinus*, *Alnus-Picea* together with *Ulmus-Quercus*, *Corylus-Taxus*, *Carpinus*, *Abies*, *Pinus-Betula.*<br>Evidence of *Pterocarya* and *Azolla*; without *Celtis*	*13*, *28*, *136*
Alversdorf<br>(only late phase recorded at Schöningen)	Predominance of *Abies*, *Picea* and *Pinus* with *Alnus;* little *Ulmus*, *Quercus*, *Tilia*, *Corylus*, *Carpinus.*<br>Vegetational succession: *Abies-Pinus-Alnus*, *Pinus-Abies-Picea*, open *Pinus*-Poaceae-Ericales vegetation.<br>Evidence of *Pterocarya* and *Azolla*	*12*, *25*
Holsteinian	Predominance of coniferous trees (*Pinus, Picea, Abies*) and *Alnus*, lower percentages of deciduous (demanding) trees.<br>Vegetational succession: *Betula-Pinus* (*Juniperus*), *Alnus-Picea* (already in the early phases), *Ulmus-Quercus*, *Corylus* (with *Taxus*), *Carpinus-Abies*, *Pinus-Picea-Betula.*<br>Evidence of *Celtis*, *Pterocarya* and *Azolla*	*128*, *137–140*

Table S4.

Provenance and amino acid data for Schöningen ostracod samples. Age for the Schöningen samples is the average of the numerical dates obtained for the D/L values and the age uncertainty is one standard deviation of all the values obtained; analytical error of ~2% is not reflected in the age calculations (see Materials and Methods for numerical age algorithm). Previously published data from Ana River (*89*) (AR07-82, AR07-108) are shown for comparison between sites with similar mean annual temperatures (Schöningen = 9.0ºC; Ana River = 9.5ºC) dated to the MIS 7/6 transition.

Specimen ID	Lab ID	Provenance	Layer	N	Species	Asp D/L	Glu D/L	Age (ka)
SCHÖ 11965	LEB 13847–13949	724/-992	4b/4c & 4c	3*	*Herpetocypris reptans, Prionocypris zenkeri*	0.330 ± 0.038	0.107 ± 0.031	114 ± 34
SCHÖ 12382	LEB 13868–13872	731/4	4b/4c	5	*Herpetocypris reptans*	0.376 ± 0.015	0.122 ± 0.013	137 ± 27
SCHÖ 12927	LEB 13873–13877	719/-996	4b	5	*Herpetocypris reptans*	0.384 ± 0.021	0.105 ± 0.100	131 ± 36
AR07-82	UAL 7583	-	-	5	*Candona* aff. *patzcuaro*	0.350 ± 0.010	0.158 ± 0.007	187
AR07-82	UAL 6608	-	-	8	*Candona* aff. *patzcuaro*	0.349 ± 0.019	0.159 ± 0.013	187
AR07-82	UAL 7432	-	-	8	*Candona* aff. *patzcuaro*	0.361 ± 0.009	0.182 ± 0.007	187
AR07-108	UAL 7584	-	-	8	*Candona* aff. *patzcuaro*	0.356 ± 0.002	0.158 ± 0.005	201
AR07-108	UAL 6616	-	-	10	*Candona* aff. *patzcuaro*	0.346 ± 0.005	0.157 ± 0.005	201
AR07-108	UAL 7433	-	-	10	*Candona* aff. *patzcuaro*	0.350 ± 0.010	0.169 ± 0.005	201

*SCHÖ 11965 originally consisted of five samples, but two of the five were judged to be contaminated and rejected for age calculation.

Table S5.

Provenance and amino acid data for Schöningen horse (*Equus mosbachensis*) tooth samples. When more than one sample per provenience is indicated, the age is the average of the numerical dates obtained for the D/L values and the age uncertainty is one standard deviation of all the values obtained; analytical error of ~2% is not reflected in the age calculations (see Materials and Methods for numerical age algorithm).

Schöningen ID	Lab ID	Provenance	Layer	N	D/L Asp	D/L Glu	Age (ka)
2715	LEB 14051	686/32	4b/4c	1	0.154	0.036	123.3
3038	LEB 14052	687/23	4b	1	0.235	0.042	194.0
3060	LEB 14054– 14055	687/24	4b	2	0.208 ± 0.017	0.039 ± 0.001	170 ± 15
5099	LEB 14056	691/14	4b	1	0.151	0.035	121.1
5681	LEB 14057	692/14	4b	1	0.155	0.035	124.2
10075	LEB 14053	711/20	4c	1	0.205	0.037	167.2
11123	LEB 14058	718/15	4c	1	0.212	0.038	173.8
11295	LEB 14059	719/13	4b/4c	1	0.184	0.036	149.2

Table S6. Provenance and amino acid data for opercula samples from comparator sites (as shown in Fig. 3): Neumark Nord 2 (*38*), Trianglen (*37*), Holsteinian horizons at Paludinenbank and Bilzingsleben, Miesenheim I, and Voigtstedt (*84*). Error terms represent mean values for the duplicate analyses of >2 individual samples per site, shown at one standard deviation. Each sample was bleached (b), with the free amino acid fraction signified by "F" and the total hydrolysable fraction by "H*" (see Materials and Methods).

Sample ID	Site	Provenance	NEaar no.	Asx D/L	Glx D/L	Ser D/L	Ala D/L	Val D/L
NN2†	Neumark Nord 2	Hauptprofil (HP) 7	4714–17; 4928–30; 5628–50; 5687–97bF	0.639 ± 0.022	0.227 ± 0.029	0.926 ± 0.042	0.265 ± 0.022	0.167 ± 0.013
NN2†	Neumark Nord 2	Hauptprofil (HP) 7	4714–17; 4928–30; 5628–50; 5687–97bH*	0.519 ± 0.025	0.157 ± 0.013	0.577 ± 0.059	0.194 ± 0.017	0.100 ± 0.017
DTBto1–4F	Trianglen‡	Interglacial deposits, associated with core P07.112	11622–4, 11654bF	0.645 ± 0.009	0.164 ± 0.001	0.925 ± 0.020	0.284 ± 0.018	0.162 ± 0.019
DTBto1–4H*	Trianglen‡	Interglacial deposits, associated with core P07.112	11622–4, 11654bH*	0.563 ± 0.008	0.143 ± 0.011	0.664 ± 0.019	0.217 ± 0.008	0.117 ± 0.005
LoBto1–4bF	Lowenbrauerei, Hohenschonhausen, Berlin	Wasserbohrung, upper depth 66, Paludinenbank	3954–6, 4762bF	0.764 ± 0.036	0.337 ± 0.044	1.012 ± 0.022	0.485 ± 0.029	0.285 ± 0.021
LoBto1–4bH*	Lowenbrauerei, Hohenschonhausen, Berlin	Wasserbohrung, upper depth 66, Paludinenbank	3954–6, 4762bH*	0.631 ± 0.036	0.251 ± 0.013	0.663 ± 0.090	0.382 ± 0.025	0.202 ± 0.012
Bil1Bto1-4bF, Bil2Bto1-4bF	Bilzingsleben	GB181200 & C171200	6179–82bF	0.776 ± 0.007	0.327 ± 0.013	1.030 ± 0.042	0.492 ± 0.007	0.258 ± 0.008
Bil1Bto1–4bH*, Bil2Bto1–4bH*	Bilzingsleben	GB181200 & C171200	6179–82bH*	0.648 ± 0.020	0.271 ± 0.009	0.730 ± 0.069	0.425 ± 0.017	0.207 ± 0.019
E22–5 GMi1Bto1–2bF	Miesenheim I	Layer 5	15153–4bF	0.784 ± 0.059	0.396 ± 0.036	0.654 ± 0.415	0.590 ± 0.010	0.333 ± 0.012
E22–5 GMi1Bto1–2bH*	Miesenheim I	Layer 5	15153–4bH*	0.747 ± 0.023	0.359 ± 0.008	0.630 ± 0.152	0.515 ± 0.006	0.290 ± 0.023
Voi6Bto1–3bF, Voi7Bto1–2bF	Voigtstedt	Unit g & h, Profile I	6807–11bF	0.830 ± 0.007	0.508 ± 0.028	0.899 ± 0.132	0.720 ± 0.007	0.427 ± 0.015
Voi6Bto1–3bH*, Voi7Bto1–2bH*	Voigtstedt	Unit g & h, Profile I	6807–11bH*	0.740 ± 0.026	0.440 ± 0.031	0.591 ± 0.081	0.611 ± 0.014	0.336 ± 0.016

†See ref. (*38*) for list of Sample IDs for Neumark Nord 2.

‡Trianglen samples were noted as not treated with NaOH, dried at room temperature.

REFERENCES AND NOTES

1. H. Thieme, Lower Palaeolithic hunting spears from Germany. *Nature* **385**, 807–810 (1997).

2. D. Leder, J. Lehmann, A. Milks, T. Koddenberg, M. Sietz, M. Vogel, U. Böhner, T. Terberger, The wooden artifacts from Schöningen's Spear Horizon and their place in human evolution. *Proc. Natl. Acad. Sci. U.S.A.* **121**, e2320484121 (2024).

3. J. Serangeli, N. J. Conard, The behavioral and cultural stratigraphic contexts of the lithic assemblages from Schöningen. *J. Hum. Evol.* **89**, 287–297 (2015).

4. J. M. Hutson, A. Villaluenga, A. García-Moreno, E. Turner, S. Gaudzinski-Windheuser, Persistent predators: Zooarchaeological evidence for specialized horse hunting at Schöningen 13II-4. *J. Hum. Evol.* **196**, 103590 (2024).

5. B. Voormolen, "Ancient hunters, modern butchers: Schöningen 13II-4, a kill-butchery site dating from the northwest European Lower Palaeolithic," thesis, Leiden University (2008).

6. M.-A. Julien, F. Rivals, J. Serangeli, H. Bocherens, N. J. Conard, A new approach for deciphering between single and multiple accumulation events using intra-tooth isotopic variations: Application to the Middle Pleistocene bone bed of Schöningen 13 II-4. *J. Hum. Evol.* **89**, 114–128 (2015).

7. T. van Kolfschoten, E. Buhrs, I. Verheijen, The larger mammal fauna from the Lower Paleolithic Schöningen Spear site and its contribution to hominin subsistence. *J. Hum. Evol.* **89**, 138–153 (2015).

8. J. M. Hutson, A. Villaluenga, A. García-Moreno, E. Turner, S. Gaudzinski-Windheuser, "A zooarchaeological and taphonomic perspective of hominin behaviour from the Schöningen 13II-4 'Spear Horizon'" in *Human Behavioural Adaptations to Interglacial Lakeshore Environments*, A. García-Moreno, J. M. Hutson, G. M. Smith, L. Kindler, E. Turner, A. Villaluenga, S. Gaudzinski-Windheuser, Eds. (RGZM-Tagungen, Band 37, Römisch-Germanisches Zentralmuseum, 2020), pp. 43–66.

9. A. García-Moreno, J. M. Hutson, A. Villaluenga, E. Turner, S. Gaudzinski-Windheuser, Connecting bones at Schöningen 13II-4 “Spear Horizon”: An analysis of site formation and human activity through faunal refitting. *Archaeol. Anthropol. Sci.* **15**, 178 (2023).

10. N. J. Conard, J. Serangeli, U. Böhner, B. M. Starkovich, C. E. Miller, B. Urban, T. van Kolfschoten, Excavations at Schöningen and paradigm shifts in human evolution. *J. Hum. Evol.* **89**, 1–17 (2015).

11. J. Serangeli, U. Böhner, T. van Kolfschoten, N. J. Conard, Overview and new results from large-scale excavations in Schöningen. *J. Hum. Evol.* **89**, 27–45 (2015).

12. B. Urban, R. Lenhard, D. Mania, B. Albrecht, Mittelpleistozän im Tagebau Schoningen. *Ldkr. Helmstedt. Z. Dtsch. Geol. Ges.* **142**, 351–372 (1991).

13. B. Urban, “Interglacial pollen records from Schöningen, northern Germany” in *The Climate of Past Interglacials*, F. Sirocko, M. Claussen, M. F. Sánchez Goñi, T. Litt, Eds. (Developments in Quaternary Science 7, Elsevier, 2007), pp. 417–444.

14. D. Mania, M. Altermann, “Das Quartär von Schöningen im nördlischen Harzvorland” in *Die Geologie der paläolithischen Fundstellen von Schöningen*, T. Terberger, S. Winghart, Eds. (Forschungen zur Urgeschichte aus dem Tagebau von Schöningen, Band 2, Römisch-Germanisches Zentralmuseum, 2015), pp. 1–190.

15. S. Toucanne, S. Zaragosi, J. F. Bourillet, P. L. Gibbard, F. Eynaud, J. Giraudeau, J. L. Turon, M. Cremer, E. Cortijo, P. Martinez, L. Rossignol, A 1.2 Ma record of glaciation and fluvial discharge from the West European Atlantic margin. *Quat. Sci. Rev.* **28**, 2974–2981 (2009).

16. J. Ehlers, A. Grube, H.-J. Stephan, S. Wansa, “Pleistocene glaciations of north Germany—New results” in *Quaternary Glaciations – Extent and Chronology. A Closer Look*, J. Ehlers, P. L. Gibbard, D. P. Hughes, Eds. (Developments in Quaternary Science 15, Elsevier, 2011), pp. 149–162.

17. T. Lauer, M. Weiss, Timing of the Saalian- and Elsterian glacial cycles and the implications for Middle – Pleistocene hominin presence in central Europe. *Sci. Rep.* **8**, 5111 (2018).

18. K. M. Cohen, P. L. Gibbard, Global chronostratigraphical correlation table for the last 2.7 million years, version 2019. *Quat. Int.* **500**, 20–31 (2019).

19. T. Litt, K.-E. Behre, K.-D. Meyer, H.-J. Stephan, S. Wansa, Stratigraphische Begriffe für das Quartär des norddeutschen Vereisungsgebietes. *E&G Quat. Sci. J.* **56**, 1–2 (2007).

20. M. R. Krbetschek, D. Degering, W. Alexowsky, Infrarot-Radiofluoreszenz-Alter (IR-RF) unter-saalezeitlicher Sedimente Mittel- und Ostdeutschlands. *Z. Dtsch. Ges. Geowiss.* **159**, 133–140 (2008).

21. J. Lang, T. Lauer, J. Winsemann, New age constraints for the Saalian glaciation in northern central Europe: Implications for the extent of ice sheets and related proglacial lake systems. *Quat. Sci. Rev.* **180**, 240–259 (2018).

22. J. Ehlers, *Das Eiszeitalter*, (Springer, ed. 2, 2020).

23. M. Geyh, H. Müller, “Palynological and geochronological study of the Holsteinian/Hoxnian/Landos interglacial” in *The Climate of Past Interglacials*, F. Sirocko, M. Claussen, M. F. Sánchez Goñi, T. Litt, Eds. (Developments in Quaternary Science 7, Elsevier, 2007), pp. 387–396.

24. C. Turner, The Eemian interglacial in north European plain and adjacent areas. *Geol. Mijnbouw* **79**, 217–231 (2000).

25. B. Urban, H. Thieme, H. Elsner, Biostratigraphische, quartärgeologische und urgeschichtliche Befunde aus dem Tagebau “Schöningen”, Ldkr. Helmstedt. *Z. Dtsch. Geol. Ges.* **139**, 123–154 (1988).

26. B. Urban, M. Sierralta, M. Frechen, New evidence for vegetation development and timing of Upper Middle Pleistocene interglacials in Northern Germany and tentative correlations. *Quat. Int.* **241**, 125–142 (2011).

27. B. Urban, T. Kasper, K. J. Krahn, T. van Kolfschoten, B. Rech, M. Holzheu, M. Tucci, A. Schwalb, Landscape dynamics and chronological refinement of the Middle Pleistocene Reinsdorf Sequence of Schöningen, NW Germany. *Quat. Res.* **114**, 148–177 (2023).

28. K. J. Krahn, B. Urban, S. Pinkerneil, D. J. Horne, M. Tucci, A. Koutsodendris, A. Schwalb, Temperature and palaeolake evolution during a Middle Pleistocene interglacial–glacial transition at the Palaeolithic locality of Schöningen, Germany. *Boreas* **53**, 504–524 (2024).

29. S. Rigterink, K. J. Krahn, B. Kotrys, B. Urban, O. Heiri, F. Turner, A. Pannes, A. Schwalb, Summer temperatures from the Middle Pleistocene site Schöningen 13 II, northern Germany, determined from subfossil chironomid assemblages. *Boreas* **53**, 525–542 (2024).

30. O. Jöris, M. Baales, "Zur Altersstellung der Schöninger Speere" in *Erkenntnisjäger: Kultur und Umwelt des frühen Menschen. Festschrift für Dietrich Mania*, M. Burdukiewicz, L. Fiedler, W.-D. Heinrich, A. Justus, E. Brühl, Eds. (Veröffentlichungen des Landesamt für Denkmalpflege und Archäologie Sachsen-Anhalt, Landesmuseum für Vorgeschichte, Band 57, 2003), pp. 281–288.

31. D. Richter, M. Krbetschek, The age of the Lower Palaeolithic occupation at Schöningen. *J. Hum. Evol.* **89**, 46–56 (2015).

32. M. Tucci, K. J. Krahn, D. Richter, T. van Kolfschoten, B. Rodríguez Álvarez, I. Verheijen, J. Serangeli, J. Lehmann, D. Degering, A. Schwalb, B. Urban, Evidence for the age and timing of environmental change associated with a Lower Palaeolithic site within the Middle Pleistocene Reinsdorf sequence of the Schöningen coal mine, Germany. *Palaeogeogr. Palaeoclimatol. Palaeoecol.* **569**, 110309 (2021).

33. M. Sierralta, M. Frechen, B. Urban, "230Th/U dating results from opencast mine Schöningen" in *Die chronologische Einordnung der paläolithischen Fundstellen von Schöningen*, K.-E. Behre, Ed. (Forschungen zur Urgeschichte aus dem Tagebau von Schöningen, Band 1, Römisch-Germanisches Zentralmuseum, 2012), pp. 143–154.

34. M. Sierralta, B. Urban, G. Linke, M. Frechen, Middle Pleistocene interglacial peat deposits from Northern Germany investigated by 230Th/U and palynology: Case studies from Wedel and Schöningen. *Z. Dtsch. Ges. Geowiss.* **168**, 373–387 (2017).

35. K. E. H. Penkman, R. C. Preece, D. R. Bridgland, D. H. Keen, T. Meijer, S. A. Parfitt, T. S. White, M. J. Collins, An aminostratigraphy for the British Quaternary based on Bithynia opercula. *Quat. Sci. Rev.* **61**, 111–134 (2013).

36. J. F. Wehmiller, A review of amino acid racemization studies in Quaternary mollusks: Stratigraphic and chronologic applications in coastal and interglacial sites, Pacific and Atlantic coasts, United States, United Kingdom, Baffin Island, and Tropical Islands. *Quat. Sci. Rev.* **1**, 83–120 (1982).

37. O. Bennike, L. Hedenäs, K. High, J. S. Korshøj, G. Lemdahl, K. Penkman, R. C. Preece, K. Rosenlund, F. A. Viehberg, New interglacial deposits from Copenhagen, Denmark: Marine isotope stage 7. *Boreas* **48**, 107–118 (2019).

38. M. J. Sier, W. Roebroeks, C. C. Bakels, M. J. Dekkers, E. Brühl, D. De Loecker, S. Gaudzinski-Windheuser, N. Hesse, A. Jagich, L. Kindler, W. J. Kuijper, T. Laurat, H. J. Mücher, K. E. H. Penkman, D. Richter, D. J. J. van Hinsbergen, Direct terrestrial-marine correlation demonstrates surprisingly late onset of the last interglacial in central Europe. *Quatern. Res.* **75**, 213–218 (2011).

39. D. Richter, M. Krbetschek, "Preliminary luminescence dating results for two Middle Palaeolithic occupations at Neumark-Nord 2" in *Multidisciplinary Studies of the Middle Palaeolithic record from Neumark-Nord (Germany), Volume 1*, S. Gaudzinski-Windheuser, W. Roebroeks, Eds. (Landesamt für Denkmalpflege und Archäologie Sachsen-Anhalt, 2014), pp. 131–136.

40. T. Schmierer, Beitrag zur Kenntnis des faunistischen und floristischen Inhalts der Berliner Paludinenbank. *Z. Dtsch. Geol. Ges.* **74**, 207–236 (1922).

41. D. Mania, “The earliest occupation of Europe: The Elbe-Saale region (Germany)” in *The Earliest Occupation of Europe* , W. Roebroeks, T. van Kolfschoten, Eds. (Analecta Praehistorica Leidensia 27, Leiden University Press, 1995), pp. 85–101.

42. K. F. Helmens, The Last Interglacial–Glacial cycle (MIS 5–2) re-examined based on long proxy records from central and northern Europe. *Quat. Sci. Rev.* **86**, 115–143 (2014).

43. T. Litt, Ed., Stratigraphie von Deutschland - Quartär. *E. G. Quat. Sci. J.* **56**, ES187005600 (2007).

44. R. Hallik, Die Vegetationsentwicklung der Holstein-Warmzeit in Nordwestdeutschland und die Altersstellung der Kieselgurlager der südlichen Lüneburger Heide. *Z. Dtsch. Geol. Gesell.* **112**, 326–333 (1960).

45. B. Menke, “Wacken, Elster-Glazial, marines Holstein-Interglazial und Wacken-Warmzeit” in *Quartär-Exkursionen in Schleswig-Holstein*, H. E. Stremme, B. Menke, Eds. (Geologisches Landesamt Schleswig-Holstein,1980), pp. 25–35.

46. J. Serangeli, B. Rodríguez-Álvarez, M. Tucci, I. Verheijen, G. Bigga, U. Böhner, B. Urban, T. van Kolfschoten, N. J. Conard, The Project Schöningen from an ecological and cultural perspective. *Quat. Sci. Rev.* **198**, 140–155 (2018).

47. C. S. Stringer, The status of *Homo heidelbergensis* (Schoetensack 1908). *Evol. Anthropol.* **21**, 101–107 (2012).

48. P. Villa, W. Roebroeks, Neandertal demise: An archaeological analysis of the modern human superiority complex. *PLOS ONE* **9**, e96424 (2014).

49. W. Roebroeks, M. Soressi, Neanderthals revised. *Proc. Natl. Acad. Sci. U.S.A.* **113**, 6372–6379 (2016).

50. S. Gaudzinski, “Monospecific or species-dominated faunal assemblages during the Middle Paleolithic in Europe” in *Transitions Before the Transition*, E. Hovers, S. L. Kuhn, Eds. (Springer, 2006), pp. 137–147.

51. W. Rendu, S. Costamagno, L. Meignen, M.-C. Soulier, Monospecific faunal spectra in Mousterian contexts: Implications for social behavior. *Quat. Int.* **247**, 50–58 (2012).

52. M. White, P. Pettitt, D. Schreve, Shoot first, ask questions later: Interpretive narratives of Neanderthal hunting. *Quat. Sci. Rev.* **140**, 1–20 (2016).

53. P. Auguste, Chasse et charognage au paléolithique moyen: l'apport du gisement de Biache-Saint Vaast (Pas-de-Calais). *Bull. de la Soc. Préhist. Fr.* **92**, 155–168 (1995).

54. P. Valensi, The archaeozoology of Lazaret Cave (Nice, France). *Int. J. Osteoarchaeol.* **10**, 357–367 (2000).

55. B. Bratlund, Taubach revisited. *Jahrb. des RGZM* **46**, 61–174 (2000).

56. C. Farizy, F. David, J. Jaubert, Eds., *Hommes et Bisons du Paléolithique Moyen à Mauran (Haute Garonne)* (Supplément à Gallia Préhistoire 30, CNRS Éditions, 1994).

57. R. Schild, Ed., *The Killing Fields of Zwoleń: A Middle Paleolithic Kill-butchery Site in Central Poland* (Institute of Archaeology and Ethnology, Polish Academy of Sciences, 2006).

58. S. Gaudzinski, W. Roebroeks, Adults only. Reindeer hunting at the Middle Palaeolithic site Salzgitter Lebenstedt, Northern Germany. *J. Hum. Evol.* **38**, 497–521 (2000).

59. K. Ruebens, G. M. Smith, H. Fewlass, V. Sinet-Mathiot, J.-J. Hublin, F. Welker, Neanderthal subsistence, taphonomy and chronology at Salzgitter-Lebenstedt (Germany): A multifaceted analysis of morphologically unidentifiable bone. *J. Quat. Sci.* **38**, 471–487 (2023).

60. S. Gaudzinski-Windheuser, L. Kindler, K. MacDonald, W. Roebroeks, Hunting and processing of straight-tusked elephants 125.000 years ago: Implications for Neanderthal behavior. *Sci. Adv.* **9**, eadd8186 (2023).

61. A.-M. Moigne, D. Barsky, "Large mammal assemblages from Lower Palaeolithic sites in France: La Caune de L'Arago, Terra-Amata, Orgnac 3 and Cagny L'Epinette" in *The Role of Early Humans in the Accumulation of European Lower and Middle Palaeolithic Bone*

Assemblages, E. Turner, S. Gaudzinski, Eds. (Monographien des Römisch-Germanisches Zentralmuseum 42, Habelt Verlag, 1999), pp. 219–235.

62. A. Rodríguez-Hidalgo, P. Saladié, A. Ollé, J. L. Arsuaga, J. M. Bermúdez de Castro, E. Carbonell, Human predatory behavior and the social implications of communal hunting based on evidence from the TD10.2 bison bone bed at Gran Dolina (Atapuerca, Spain). *J. Hum. Evol.* **105**, 89–122 (2017).

63. S. Gaudzinski-Windheuser, L. Kindler, Research perspectives for the study of Neanderthal subsistence strategies based on the analysis of archaeological assemblages. *Quat. Int.* **247**, 59–68 (2012).

64. G. C. Frison, "Prehistoric, plains-mountain, large-mammal, communal hunting strategies" in *The Evolution of Human Hunting*, M. H. Nitecki, D. V. Nitecki, Eds. (Plenum, 1987), pp. 177–223.

65. E. Morin, D. Bird, B. Winterhalder, R. Bleige Bird, Why do humans hunt cooperatively? Ethnohistoric data reveal contexts, advantages, and evolutionary importance of communal hunting. *Curr. Anthropol.* **65**, 876–921 (2024).

66. P. E. Hare, R. M. Mitterer, Non-protein amino acids in fossil shells. *Carnegie I. Wash.* **65**, 236–364 (1967).

67. P. E. Hare, R. M. Mitterer, Laboratory simulation of amino acid diagenesis in fossils. *Carnegie I. Wash.* **67**, 205–208 (1969).

68. U. Böhner, J. Serangeli, P. Richter, The Spear Horizon: First spatial analysis of the Schöningen site 13 II-4. *J. Hum. Evol.* **89**, 202–213 (2015).

69. B. Urban, K. J. Krahn, T. Kasper, A. García-Moreno, J. M. Hutson, A. Villaluenga, E. Turner, S. Gaudzinski-Windheuser, D. Farghaly, M. Tucci, A. Schwalb, Spatial interpretation of high-resolution environmental proxy data of the Middle Pleistocene Palaeolithic faunal kill site Schöningen 13 II-4, Germany. *Boreas* **52**, 440–458 (2023).

70. G. A. Sykes, M. J. Collins, D. I. Walton, The significance of a geochemically isolated intracrystalline organic fraction within biominerals. *Org. Geochem.* **23**, 1039–1065 (1995).

71. K. E. H. Penkman, “Amino acid geochronology: A closed system approach to test and refine the UK model,” thesis, University of Newcastle (2005).

72. K. E. H. Penkman, R. C. Preece, D. H. Keen, D. Maddy, D. C. Schreve, M. J. Collins, Testing the aminostratigraphy of fluvial archives: The evidence from intra-crystalline proteins within freshwater shells. *Quat. Sci. Rev.* **26**, 2958–2969 (2007).

73. K. E. H. Penkman, D. S. Kaufman, D. Maddy, M. J. Collins, Closed-system behaviour of the intra-crystalline fraction of amino acids in mollusc shells. *Quat. Geochronol.* **3**, 2–25 (2008).

74. P. J. Hearty, M. J. O’Leary, D. S. Kaufman, M. C. Page, J. Bright, Amino acid geochronology of individual foraminifer (*Pulleniatina obliquiloculata*) tests, north Queensland margin, Australia: A new approach to correlating and dating Quaternary tropical marine sediment cores. *Paleoceanography* **19**, doi.org/10.1029/2004PA001059 (2004).

75. D. S. Kaufman, W. F. Manley, A new procedure for determining DL amino acid ratios in fossils using reverse phase liquid chromatography. *Quat. Sci. Rev.* **17**, 987–1000 (1998).

76. R. L. Hill, Hydrolysis of proteins. *Adv. Protein Chem.* **20**, 37–107 (1965).

77. J. L. Bada, M.-Y. Shou, E. H. Man, R. A. Schroeder, Decomposition of hydroxy amino acids in foraminiferal tests; kinetics, mechanism and geochronological implications. *Earth Planet. Sci. Lett.* **41**, 67–76 (1978).

78. K. E. H. Penkman, R. C. Preece, D. R. Bridgland, D. H. Keen, T. Meijer, S. A. Parfitt, T. S. White, M. J. Collins, A chronological framework for the British Quaternary based on Bithynia opercula. *Nature* **476**, 446–449 (2011).

79. R. C. Preece, K. E. H. Penkman, New faunal analyses and amino acid dating of the Lower Palaeolithic site at East Farm, Barnham, Suffolk. *Proc. Geol. Assoc.* **116**, 363–377 (2005).

80. G. H. Miller, J. Mangerud, Aminostraigraphy of European interglacial deposits. *Quat. Sci. Rev.* **4**, 215–278 (1985).

81. D. Q. Bowen, *A Revised Correlation of Quaternary Deposits in the British Isles* (Geological Society Special Report 23, Geological Society of London, 1999).

82. M. Meyer, E. Palkopoulou, S. Baleka, M. Stiller, K. E. H. Penkman, K. W. Alt, Y. Ishida, D. Mania, S. Mallick, T. Meijer, H. Meller, S. Nagel, B. Nickel, S. Ostritz, N. Rohland, K. Schauer, T. Schüler, A. L. Roca, D. Reich, B. Shapiro, M. Hofreiter, Palaeogenomes of Eurasian straight-tusked elephants challenge the current view of elephant evolution. *eLife* **6**, e25413 (2017).

83. E. Turner, *Miesenheim I: Excavations at a Lower Palaeolithic Site in the Central Rheinland of Germany* (Monographien des Römisch-Germanisches Zentralmuseum 44, 2000).

84. L. C. Maul, M. Stebich, P. Frenzel, U. Hambach, T. Henkel, L. Katzschmann, F. Kienast, S. Meng, K. Penkman, C. Rolf, M. Thomas, R.-D. Kahlke, Age and palaeoenvironment of the enigmatic Arternian Interglacial — evidence from the Muschelton at Voigtstedt/Hackelsberg (Thuringia, Central Germany). *Palaeogeogr. Palaeoclimatol. Palaeoecol.* **386**, 68–85 (2013).

85. D. S. Kaufman, "Amino acid racemization in ostracodes" in *Perspectives in Amino Acids and Protein Geochemistry*, G. A. Goodfriend, M. J. Collins, M. L. Fogel, S. A. Macko, J. F. Wehmiller, Eds. (Oxford University Press, 2000), pp. 145–160.

86. R. Lafont, G. Périnet, F. Bazile, N. Icole, Racémisation d'acides aminés d'ossements fossiles du Paléolithique supérieur languedocien. *C. R. Acad. Sci. II B* **299**, 447–450 (1984).

87. E. Marzin, Essai de normalisation du protocole d'analyse des taux de racémisation des acides aminés: Applications a la datation d'ossements fossiles. *Travaux du LAPMO* **8**, 167–178 (1990).

88. G. A. Goodfriend, Patterns of racemization and epimerization of amino acids in land snail shells over the course of the Holocene. *Geochim. Cosmochim. Acta* **55**, 293–302 (1991).

89. J. Bright, D. S. Kaufman, Amino acid racemization in lacustrine ostracodes, part I: Effect of oxidizing pre-treatments on amino acid composition. *Quat. Geochronol.* **6**, 154–173 (2011).

90. J. E. Ortiz, T. Torres, A. Pérez-González, Amino acid racemization in four species of ostracodes: Taxonomic, environmental, and microstructural controls. *Quat. Geochronol.* **16**, 129–143 (2013).

91. J. E. Ortiz, T. Torres, R. Julià, A. Delgado, F. J. Llamas, V. Soler, J. Delgado, Numerical dating algorithms of amino acid racemization ratios from continental ostracodes. Application to Guadix-Baza Basin (southern Spain). *Quat. Sci. Rev.* **23**, 717–730 (2004).

92. D. S. Kaufman, Temperature sensitivity of aspartic and glutamic acid racemization in the foraminifera Pulleniatina. *Quat. Geochronol.* **1**, 188–207 (2006).

93. T. Torres, J. F. Llamas, L. Canoira, P. García-Alonso, "Aspartic acid racemization and protein preservation in the dentine of Pleistocene European bear teeth" in *Perspectives in Amino Acids and Protein Geochemistry*, G. A. Goodfriend, M. J. Collins, M. L. Fogel, S. A. Macko, J. F. Wehmiller, Eds. (Oxford University Press, 2000), pp. 349–355.

94. T. Torres, J. F. Llamas, L. Canoira, J. E. Ortiz, M. A. García de la Morena, R. Juliá, Aspartic acid based aminostratigraphy of Spanish Ursus deningeri von Reich. and Ursus spelaeus Ros.-Hein. localities. *Beitr. Paläontol.* **25**, 177–182 (2000).

95. T. Torres, J. E. Ortiz, M. J. García, J. F. Llamas, L. Canoira, M. A. de la Morena, R. Juliá, Geochemical evolution of amino acids in dentine of Pleistocene bears. *Chirality* **13**, 517–521 (2001).

96. T. de Torres, J. E. Ortiz, F. J. Llamas, L. Canoira, R. Juliá, M. J. García-Martínez, Bear dentine aspartic acid racemization analysis: A proxy for the dating of Pleistocene cave infills. *Archeometry* **44**, 417–426 (2002).

97. L. Canoira, M.-J. García-Martínez, J. F. Llamas, J. E. Ortiz, T. Torres, Kinetics of amino acid racemization (epimerization) in the dentine of fossil and modern bear teeth. *Int. J. Chem. Kinet.* **35**, 576–591 (2003).

98. T. Torres, J. E. Ortiz, E. Fernández, E. Arroyo-Pardo, R. Grün, A. Pérez-González, Aspartic acid racemization as a dating tool for dentine: A reality. *Quat. Geochronol.* **22**, 43–56 (2014).

99. C. V. Murray-Wallace, Aminostratigraphy of Quaternary coastal sequences in southern Australia—An overview. *Quat. Int.* **26**, 69–86 (1995).

100. M. Huuse, H. Lykke-Andersen, Overdeepened Quaternary valleys in the eastern Danish North Sea: Morphology and origin. *Quat. Sci. Rev.* **19**, 1233–1253 (2000).

101. W. Stackebrandt, A. O. Ludwig, S. Ostaficzuk, Base of Quaternary deposits of the Baltic Sea depression and adjacent areas (map 2). Neogeodynamica baltica IGCP-Project 346. *Brandenburgische Geowiss. Beitr.* **8**, 13–19 (2001).

102. M. C. Stahlschmidt, C. E. Miller, B. Ligouis, P. Goldberg, F. Berna, B. Urban, N. J. Conard, The depositional environments of Schöningen 13 II-4 and their archaeological implications. *J. Hum. Evol.* **89**, 71–91 (2015).

103. E. Turner, J. Hutson, A. Villaluenga, A. García Moreno, S. Gaudzinski-Windheuser, Bone staining in waterlogged deposits: A preliminary contribution to the interpretation of near-shore find accumulation at the Schöningen 13II-4 'Spear-Horizon' site, Lower Saxony, Germany. *Hist. Biol.* **30**, 767–773 (2017).

104. A. García-Moreno, J. M. Hutson, A. Villaluenga, E. Turner, S. Gaudzinski-Windheuser, A detailed analysis of the spatial distribution of Schöningen 13II-4 'Spear Horizon' faunal remains. *J. Hum. Evol.* **152**, 102947 (2021).

105. C. Peters, T. van Kolfschoten, The site formation history of Schöningen 13II-4 (Germany): Testing different models of site formation by means of spatial analysis, spatial statistics and orientation analysis. *J. Archaeol. Sci.* **114**, 105067 (2020).

106. J. Jouzel, V. Masson-Delmotte, O. Cattani, G. Dreyfus, S. Falourd, G. Hoffmann, B. Minster, J. Nouet, J. M. Barnola, J. Chappellaz, H. Fischer, J. C. Gallet, S. Johnsen, M. Leuenberger, L. Loulergue, D. Luethi, H. Oerter, F. Parrenin, G. Raisbeck, D. Raynaud, A. Schilt, J. Schwander, E. Selmo, R. Souchez, R. Spahni, B. Stauffer, J. P. Steffensen, B.

Stenni, T. F. Stocker, J. L. Tison, M. Werner, E. W. Wolff, Orbital and millennial Antarctic climate variability over the past 800,000 years. *Science* **317**, 793–796 (2007).

107. Past Interglacials Working Group of PAGES, Interglacials of the last 800,000 years. *Rev. Geophys.* **54**, 162–219 (2016).

108. L. B. Railsback, P. L. Gibbard, M. J. Head, N. R. G. Voarintsoa, S. Toucanne, An optimized scheme of lettered marine isotope substages for the last 1.0 million years, and the climatostratigraphic nature of isotope stages and substages. *Quat. Sci. Rev.* **111**, 94–106 (2015).

109. H. Thieme, Ed., *Die Schöninger Speere: Mensch und Jagd vor 400 000 Jahren* (Konrad Theiss Verlag, 2007).

110. J. Lang, J. Winsemann, D. Steinmetz, U. Polom, L. Pollok, U. Böhner, J. Serangeli, C. Brandes, A. Hampel, S. Winghart, The Pleistocene of Schöningen, Germany: A complex tunnel valley fill revealed from 3D subsurface modeling and shear wave seismics. *Quat. Sci. Rev.* **39**, 86–105 (2012).

111. B. Urban, Palynological evidence of younger Middle Pleistocene Interglacials (Holsteinian, Reinsdorf, Schöningen) in the Schöningen open cast lignite mine (eastern Lower Saxony/ Germany). *Meded. Rijks Geol. Dienst* **52**, 175–186 (1995).

112. B. Urban, M. Sierralta, "New palynological evidence and correlation of Early Palaeolithic sites Schöningen 12 B and 13 II, Schöningen open lignite mine" in *Die chronologische Einordnung der paläolithischen Fundstellen von Schöningen*, K.-E. Behre, Ed. (Forschungen zur Urgeschichte aus dem Tagebau von Schöningen, Band 1, Römisch-Germanisches Zentralmuseum, 2012), pp. 77–96.

113. S. Wansa, J. Strahl, I. Rappsilber, Eds., *Zur Geologie des Ummendorfer Kessels im oberen Allertal – Forschungsbohrung Ummendorf 1/2012* (Mitteilungen zu Geologie und Bergwesen von Sachsen-Anhalt 20, Landesamt für Geologie und Bergwesen von Sachsen-Anhalt, Halle, 2019).

114. K. J. Krahn, M. Tucci, B. Urban, J. Pilgrim, P. Frenzel, I. Soulié-Märsche, A. Schwalb, Aquatic and terrestrial proxy evidence for Middle Pleistocene paleolake and lake-shore development at two Lower Palaeolithic sites of Schöningen, Germany. *Boreas* **50**, 723–745.

115. A. Kunz, B. Urban, S. Tsukamoto, Chronological investigations of Pleistocene interglacial, glacial and aeolian deposits from Schöningen (Germany) using post-IR IRSL dating and pollen analysis. *Z. Dtsch. Ges. Geowiss.* **168**, 81–104 (2017).

116. X. Rui, B. Li, Y. Guo, The effect of residual signal on dose measurements using MET-pIRIR signals from K-feldspar. *Quat. Geochronol.* **58**, 101065 (2020).

117. J. Zhang, B. Guralnik, S. Tsukamoto, C. Ankjærgaard, T. Reimann, The bleaching limits of IRSL signals at various stimulation temperatures and their potential inference of the pre-burial light exposure duration. *Front. Earth Sci.* **10**, 933131 (2023).

118. M. Stebich, D. Höfer, J. Mingram, N. Nowaczyk, J. Rohrmüller, J. Mrlina, H. Kämpf, A contribution towards the palynostratigraphical classification of the Middle Pleistocene in Central Europe: The pollen record of the Neualbenreuth Maar, northeastern Bavaria (Germany). *Quat. Sci. Rev.* **250**, 106681 (2020).

119. J. Serangeli, U. Böhner, H. Haßmann, N. J. Conard, “Die pleistozänen Fundstelllen in Schöningen — eine Einführung” in *Die chronologische Einordnung der paläolithischen Fundstellen von Schöningen*, K.-E. Behre, Ed. (Forschungen zur Urgeschichte aus dem Tagebau von Schöningen, Band 1, Römisch-Germanisches Zentralmuseum, 2012), pp. 1–22.

120. K. M. Towe, “Preserved organic ultrastructure: An unreliable indicator for Paleozoic amino acid biogeochemistry” in *Biogeochemistry of Amino Acids*, P. E. Hare, T. C. Hoering, K. King, Eds. (Wiley, 1980), pp. 65–74.

121. R. M. Mitterer, Ages and diagenetic temperatures of Pleistocene deposits of Florida based on isoleucine epimerization in Mercenaria. *Earth Planet. Sci. Lett.* **28**, 275–282 (1975).

122. P. J. Hearty, G. H. Miller, C. E. Stearns, B. J. Szabo, Aminostratigraphy of Quaternary shorelines in the Mediterranean basin. *Geol. Soc. Am. Bull.* **97**, 850–858 (1986).

123. P. J. Hearty, D. S. Kaufman, Whole-rock aminostratigraphy and Quaternary sea-level history of the Bahamas. *Quatern. Res.* **54**, 163–173 (2000).

124. J. E. Ortiz, I. Gutiérrez-Zugasti, T. Torres, M. González-Morales, Y. Sánchez-Palencia, Protein diagenesis in Patella shells: Implications for amino acid racemisation dating. *Quat. Geochronol.* **27**, 105–118 (2015).

125. G. H. Miller, J. W. Magee, B. J. Johnson, M. L. Fogel, N. A. Spooner, M. T. McCulloch, L. K. Ayliffe, Pleistocene extinction of *Genyornis newtoni*: Human impact on Australian megafauna. *Science* **283**, 205–208 (1999).

126. P. Harting, De bodem van het Eemdal. *Versl. Koninklijk. Acad. Wet., Afdeling Natuurkd* **8**, 282–290 (1874).

127. A. Jessen, V. Milthers, Stratigraphical and paleontological studies of interglacial freshwater deposits in Jutland and Northwest Germany. *Danm. Geol. Unders.* **48**, 1–379 (1928).

128. H. Müller, Pollenanalytische Untersuchungen und Jahresschichtenzählungen an der Eem-zeitlichen Kieselgur von Bispingen/Luhe. *Geol. Jahrb.* **A21**, 149–169 (1974).

129. B. Menke, R. Tynni, Das Eeminterglazial und das Frühweichselglazial von Redderstall/Dithmarschen und ihre Bedeutung für die mitteleuropäische Jungpleistozän-Gliederung. *Geol. Jahrb.* **A76**, 3–120 (1984).

130. T. Litt, Pollenanalytische Untersuchungen zur Vegetations- und Klimaentwicklung während des Jungpleistozäns in den Becken von Gröbern und Grabschütz. *Altenburger Naturwiss. Forsch.* **5**, 92–105 (1990).

131. H.-J. Stephan, B. Urban, G. Lüttig, B. Menke, M. Sierralta, Eds., *Palynologische, petrographische und geochronologische Untersuchungen an Ablagerungen der Leck-Warmzeit (spätes Mittelpleistozän) und begleitender Sedimente* (Geologisches Jahrbuch Reihe A, Band A 160, Schweizbart'sche Verlagsbuchhandlung, 2012).

132. K. Erd, Pollenanalytische Gliederung des mittelpleistozänen Richtprofils Pritzwalk-Prignitz. *E&G Quat. Sci. J.* **16**, 252–253 (1965).

133. B. Menke, Beitrage zur Biostratigraphie des Mittelpleistozans in Norddeutschland (Pollenanalytische Untersuchungen aus Westholstein). *Meyniana* **18**, 35–42 (1968).

134. K. Erd, Pollenanalytical classification of the Middle Pleistocene in the German Democratic Republic. *Palaeogeogr. Palaeoclimatol. Palaeoecol.* **8**, 129–145 (1970).

135. K. Erd, Vegetationsentwicklung und Biostratigraphie der Dömnitz-Warmzeit (Fuhne/Saale 1) im Profil von Pritzwalk/Prignitz. *Abh. Zentr. Geol. Inst.* **18**, 9–48 (1973).

136. J. Strahl, "Ergebnisse palynologischer Untersuchungen an der Forschungsbohrung Ummerndorf 1/2012 and Vergleich mit anderen pollenstratigraphischen Untersuchungen im oberen Allertal" in *Zur Geologie des Ummendorfer Kessels im oberen Allertal: Forschungsbohrung Ummendorf 1/2012*, S. Wansa, J. Strahl, I. Rappsilber, Eds. (Mitteilungen zu Geologie und Bergwesen von Sachsen-Anhalt 20, Landesamt für Geologie und Bergwesen Sachsen-Anhalt, 2019), pp. 41–92.

137. J. Geikie, *The Great Ice Age and its Relationship to the Antiquity of Man* (Edward Stanford, ed. 3, 1894).

138. G. Linke, R. Hallik, Die pollenanalytischen Ergebnisse der Bohrungen Hanmburg-Dockenhuden (qho 4), Wedel, (qho 2) und Hamburg-Billbrock. *Geol. Jahrb.* **A138**, 169–184 (1993).

139. H. Müller, H.-C. Höfle, Die Holstein-Interglazialvorkommen bei Bossel westlich von Stade und Wanhöden nördlich Bremerhaven. *Geol. Jahrb.* **A134**, 71–116 (1994).

140. L. Eißmann, T. Litt, S. Wansa, "Elsterian and Saalian deposits in their type area in central Germany" in *Glacial Deposits in North-East Europe*, J. Ehlers, S. Kozarski, P. L. Gibbard, Eds. (Balkema, 1995), pp. 439–464.
